# Implementation of Online Behavior Modification Techniques in the Management of Chronic Musculoskeletal Pain: A Systematic Review and Meta-Analysis

**DOI:** 10.3390/jcm11071806

**Published:** 2022-03-24

**Authors:** Ferran Cuenca-Martínez, Laura López-Bueno, Luis Suso-Martí, Clovis Varangot-Reille, Joaquín Calatayud, Aida Herranz-Gómez, Mario Romero-Palau, José Casaña

**Affiliations:** 1 Exercise Intervention for Health Research Group (EXINH-RG), Department of Physiotherapy, University of Valencia, 46010 Valencia, Spain; ferran.cuenca@uv.es (F.C.-M.); clovis.varangotreille@gmail.com (C.V.-R.); joaquin.calatayud@uv.es (J.C.); aidahergo10@gmail.com (A.H.-G.); jose.casana@uv.es (J.C.); 2 Department of Psychology, University of Valencia, 46010 Valencia, Spain; mromeropalau1998@gmail.com

**Keywords:** telerehabilitation, behavioral modification techniques, pain intensity, chronic pain

## Abstract

Purpose: The main aim of this systematic review and meta-analysis (MA) was to assess the effectiveness of online behavior modification techniques (e-BMT) in the management of chronic musculoskeletal pain. Methods: We conducted a search of Medline (PubMed), Cumulative Index to Nursing and Allied Health Literature (CINAHL), Web of Science, APA PsychInfo, and Psychological and Behavioral Collections, from inception to the 30 August 2021. The main outcome measures were pain intensity, pain interference, kinesiophobia, pain catastrophizing and self-efficacy. The statistical analysis was conducted using RStudio software. To compare the outcomes reported by the studies, we calculated the standardized mean difference (SMD) over time and the corresponding 95% confidence interval (CI) for the continuous variables. Results: Regarding pain intensity (vs. usual care/waiting list), we found a statistically significant trivial effect size in favor of e-BMT (n = 5337; SMD = −0.17; 95% CI −0.26, −0.09). With regard to pain intensity (vs. in-person BMT) we found a statistically significant small effect size in favor of in-person BMT (n = 486; SMD = 0.21; 95%CI 0.15, 0.27). With respect to pain interference (vs. usual care/waiting list) a statistically significant small effect size of e-BMT was found (n = 1642; SMD = −0.24; 95%CI −0.44, −0.05). Finally, the same results were found in kinesiophobia, catastrophizing, and self-efficacy (vs. usual care/waiting list) where we found a statistically significant small effect size in favor of e-BMT. Conclusions: e-BMT seems to be an effective option for the management of patients with musculoskeletal conditions although it does not appear superior to in-person BMT in terms of improving pain intensity.

## 1. Introduction

The serious health crisis the world is currently experiencing as a result of coronavirus disease 2019 (COVID-19) is affecting virtually all social and professional spheres [1]. At the clinical level, conventional rehabilitation consultations have had to be suspended, and many patients have had to interrupt their standard or conventional therapy (face to face). A small percentage of patients have begun undergoing therapy through telematic channels [1]. Although is still too early to determine the actual percentage of clinicians who have incorporated telerehabilitation (TR) into their portfolio of services, we suspect that there have been few. TR is defined as the implementation of a virtual, technology-based clinical-healthcare intervention in order to deliver care at a distance [2].

The person-centered model of care encompasses a number of dimensions in which the therapist–patient alliance, behavioral analysis, the patient as a whole, patient empowerment and finally the therapist’s perspective are included [3]. It involves a range of tools in the rehabilitation of patients, with behavior change or modification techniques (BMT) being one of them [3]. According to Pear and Martin [4], BMT are techniques where learning principles are systematically applied to assess, change and/or improve people’s covert and overt behaviors to enhance the solution of practical problems. BMT includes a variety of psychological techniques, such as: goal and target setting, self-monitoring, cognitive restructuring, motivational interviewing, dissociation, self-reinforcement, problem solving, coping skills training, behavior contract, establishment of reinforcement contingencies, or general instruction on how to perform behaviors [5,6,7,8,9,10]. The fundamental difference between BMT and e-BMT is that the latter is carried out through TR, i.e., via telecommunication in order to be able to intervene remotely. It should be noted that implementing e-BMT is not just the same intervention as conventional BMT but has a number of considerations that need to be taken into account. In the scientific literature, barriers to be considered have been raised and are of great interest: the lack of legal regulations, technical limitations such as the bandwidth required for the transmission of data, images and sound, training in the use of new technologies, issues associated with the payment of insurers and significant changes in the management and redesign of existing care models [11,12].

Patients with chronic musculoskeletal pain have been one of the subsets of patients most affected by COVID-19 due to lack of access to treatment for their clinical conditions [13]. Failure to treat these patients can have very serious socio-health consequences [14]. Strategies need to be put in place to curb the impact of the COVID-19 pandemic on patients with persistent musculoskeletal pain. TR could be an effective way to counteract the burden of the COVID-19 pandemic in patients with chronic musculoskeletal pain [15,16]. Pain management has been extensively studied in the current state of the art. We can find different clinical interventions for the management of pain patients. For example, treatments based on therapeutic exercise [17], manual therapy [18], pharmacology [19], combined [20], among many others. Educational interventions aim to change maladaptive behaviors, dysfunctional thoughts, beliefs, ideas, cognitions in general, as well as to improve moods and increase motivation levels in order to improve problem solving in the lives of pain patients [21]. Educational interventions can improve levels of self-efficacy as well as modify behaviors by increasing levels of therapeutic exercise as well as levels of adherence to have an impact on the neurophysiology of pain [22], because we know the full implications of exercise on pain processing [23]. Interventions based on TR offer us the option of being able to improve indirect aspects in a delocalized manner, which is why we believe it is important to study and clinically evaluate them. Some previous systematic reviews have assessed the effect of telerehabilitation based on BMT on variables such as pain intensity, disability, disease impact, physical function, pain-related fear of movement, and psychological distress [24,25,26,27] showing promising results.

It is therefore that the main aim of this systematic and meta-analysis was to assess the effectiveness of online BMT (e-BMT) in the management of patients with chronic musculoskeletal pain.

## 2. Materials and Methods

This systematic review and meta-analysis was performed according to the Preferred Reporting Items for Systematic Reviews and Meta-analysis (PRISMA) 2020 statement actualized by Page et al. [28] (Appendix A). This systematic review was registered prospectively in an international database PROSPERO where it can be accessed (CRD42021276104).

### 2.1. Inclusion Criteria

The selection criteria used in this systematic review and meta-analysis were based on methodological and clinical factors, such as the Population, Intervention, Control, Outcomes, and Study design (PICOS) described by Stone [29].

#### 2.1.1. Population

The participants selected for the studies were patients older than 18 years with any kind of chronic musculoskeletal disorder. The participants’ gender was irrelevant. We excluded patients with musculoskeletal pain due to oncologic or traumatic process. 

#### 2.1.2. Intervention and Control

The intervention was e-BMT applied through a technology device (Website, online, telephone or mobile application). The intervention could be applied alone or embedded with another treatment, only if the control group contains only the additional treatment. Control group could be usual care, waiting list, no intervention or in-person equivalent BMT.

#### 2.1.3. Outcomes

The measures used to assess the results were pain intensity, pain interference, kinesiophobia, pain catastrophizing and self-efficacy. Time of measurement was restrained to post-treatment results. 

#### 2.1.4. Study Design

We only included randomized studies (randomized controlled trials (RCTs) or randomized parallel design-controlled trials) given the amount of literature available in this area.

### 2.2. Search Strategy

The search for studies was performed using Medline (PubMed), Cumulative Index to Nursing and Allied Health Literature (CINAHL), Web of Science, APA PsychInfo, and Psychological and Behavioral Collections, from inception to the 30 August 2021. The search strategy used in Medline (PubMed) combined medical subject headings (MeSH) and non-MeSH terms, adding a Boolean operator (OR and/or AND) to combine them. Several terms we used were as follows: “ehealth”, “mhealth”, “remote treatment”, “digital treatment”, “Mobile Applications”, “Web”, “Software”, “Online”, “Telephone”, “Cell phone”, “eTherapy”, “Internet”; “Telerehabilitation”, “Interned-Based Intervention”, “Telemedicine”, “Behavioral Modification Techniques”, “Chronic Pain”, “Pain”, “RCT” or “Randomized controlled trial”. 

The search strategy was adapted to other electronic databases. In addition, we manually checked the reference of the studies included in the review and we checked the studies included on systematic review related to this topic. The search was also adapted and performed in Google Scholar due to its capacity to search for relevant articles and grey literature [30,31]. No restrictions were applied to any specific language as recommended by the international criteria [32]. The different search strategies used are detailed in Appendix B.

Two independent reviewers conducted the search using the same methodology, and the differences were resolved by consensus moderated by a third reviewer. We used Rayyan software to organize studies, assess studies for eligibility and remove duplicates [33].

### 2.3. Selection Criteria and Data Extraction

The two phases of studies selection (title/abstract screening and full-text evaluation) were realized by two independent reviewers. First, they assessed the relevance of the studies regarding the study questions and aims, based on information from the title, abstract and keywords of each study. If there was no consensus or the abstracts did not contain sufficient information, the full text was reviewed. In the second phase of the analysis, the full text was used to assess whether the studies met all the inclusion criteria. Differences between the two independent reviewers were resolved by a consensus process moderated by a third reviewer [34]. Data described in the results were extracted by means of a structured protocol that ensured that the most relevant information was obtained from each study [35].

### 2.4. Risk of Bias and Methodological Quality Assessment

The Risk Of Bias 2 (RoB 2) tool was used to assess randomized trials [36]. It covers a total of five domains: (1) Bias arising from the randomization process, (2) Bias due to deviations from the intended interventions, (3) Bias due to missing outcome data, (4) Bias in measurement of the outcome, (5) Bias in selection of the reported result. The study will be categorized has having (a) low risk of bias if all domains shown low risk of bias, (b) some concerns if one domain is rated with some concerns without any with high risk of bias, and (c) high risk of bias, if one domain is rated as having high risk of bias or multiple with some concerns. 

The studies’ methodological quality was assessed using the PEDro scale [37], which assesses the internal and external validity of a study and consists of 11 criteria: (1) specified study eligibility criteria, (2) random allocation of patients, (3) concealed allocation, (4) measure of similarity between groups at baseline, (5) patient blinding, (6) therapist blinding, (7) assessor blinding, (8) fewer than 15% dropouts, (9) intention-to-treat analysis, (10) intergroup statistical comparisons, and (11) point measures and variability data. The methodological criteria were scored as follows: yes (1 point), no (0 points), or do not know (0 points). The PEDro score for each selected study provided an indicator of the methodological quality (9–10 = excellent; 6–8 = good; 4–5 = fair; 3–0 = poor) [38]. We used the data obtained from the PEDro scale to map the results of the quantitative analyses. 

Two independent reviewers examined the quality and the risk of bias of all the selected studies using the same methodology. Disagreements between the reviewers were resolved by consensus with a third reviewer. The concordance between the results (inter-rater reliability) was measured using Cohen’s kappa coefficient (κ) as follows: (1) κ > 0.7 indicated a high level of agreement between assessors; (2) κ = 0.5–0.7 indicated a moderate level of agreement; and (3) κ < 0.5 indicated a low level of agreement) [39].

### 2.5. Certainty of Evidence 

The certainty of evidence analysis was based on classifying the results into levels of evidence according to the Grading of Recommendations, Assessment, Development and Evaluation (GRADE) framework, which is based on five domains: study design, imprecision, indirectness, inconsistency and publication bias [40]. The assessment of the five domains was conducted according to GRADE criteria [41,42]. Evidence was categorized into the following four levels accordingly: (a) *High quality.* Further research is very unlikely to change our confidence in the effect estimate. All five domains are also met; (b) *Moderate quality.* Further research is likely to have an important impact on our confidence in the effect estimate and might change the effect estimate. One of the five domains is not met; (c) *Low quality.* Further research is very likely to have a significant impact on our confidence in the effect estimate and is likely to change the estimate. Two of the five domains are not met; and (d) *Very low quality.* Any effect estimates highly uncertain. Three of the five domains are not met [41,42].

For the risk of bias domain, the recommendations were downgraded one level in the event there was an uncertain or high risk of bias and serious limitations in the effect estimate (more that 25% of the participants were from studies with high risk of bias, as measured by the RoB2 scale). In terms of inconsistency, the recommendations were downgraded one level when the point estimates varied widely among studies, the confidence intervals showed minimal overlap or when the I^2^ was substantial or large (greater than 50%). In regard to indirectness, domain recommendations were downgraded when severe differences in interventions, study populations or outcomes were found (the recommendations were downgraded in the absence of direct comparisons between the interventions of interest or when there are no key outcomes, and the recommendation is based only on intermediate outcomes or if more than 50% of the participants were outside the target group). For the imprecision domain, the recommendations were downgraded one level if there were fewer than 300 participants for the continuous data [43]. Finally, the recommendations were downgraded due to the strong influence of publication bias if the results changed significantly after adjusting for publication bias.

### 2.6. Data Synthesis and Analysis

The statistical analysis was conducted using *RStudio* software (RStudio, PBC, Boston, MA) according to the guide from Harrer et al. [44]. To compare the outcomes reported by the studies, we calculated the standardized mean difference (SMD) over time and the corresponding 95% confidence interval (CI) for the continuous variables. The statistical significance of the pooled SMD was examined as Hedges’ g to account for a possible overestimation of the true population effect size in the small studies [45]. The estimated SMDs were interpreted as described by Hopkins et al. [46], that is, we considered that an SMD of 4.0 represented an extremely large clinical effect, 2.0–4.0 represented a very large effect, 1.2–2.0 represented a large effect, 0.6–1.2 represented a moderate effect, 0.2–0.6 represented a small effect and 0.0–0.2 represented a trivial effect. If necessary, CI and standard error (SE) where converted in standard deviation (SD) using the formulas recommended by the Cochrane Handbook for Systematic Reviews of Interventions version 6.2: *SD = √(N) ∗ (upper limit − lower limit)/3.92* and *SD = √(N) ∗ SE*, respectively [47].

We used the same inclusion criteria for the systematic review and the meta-analysis and included three additional criteria: (1) In the results, there was detailed information regarding the comparative statistical data of the exposure factors, therapeutic interventions, and treatment responses; (2) the intervention was compared with a similar control group; and (3) data on the analyzed variables were represented in at least three studies. 

Since we pooled different treatments, we could not assume that there was a unique true effect. So, we anticipated between-study heterogeneity and used a random-effects model to pool effect sizes. In order the calculate the heterogeneity variance τ^2^, we used the Restricted Maximum Likelihood Estimator as recommended for continuous outcomes [48,49]. To calculate the confidence interval around the pooled effect, we used Knapp-Hartung adjustments [50,51]. 

In order to pool the catastrophizing variable and the different subscales of the Pain Catastrophizing scale [52], we ran a subgroup analysis using fixed-effects (plural) model [53]. First, we pooled effect sizes in each subgroup (Pain catastrophizing or other catastrophizing overall score, Helplessness, Magnification and Rumination) using a random-effects model. Finally, we used a fixed-effect model to pool the pooled effects from the different subgroups. 

We estimated the degree of heterogeneity among the studies using Cochran’s Q statistic test (a *p*-value < 0.05 was considered significant), the inconsistency index (I^2^) and the prediction interval (PI) based on the between-study variance τ^2^ [46]. The Cochran’s Q test allows us to assess the presence of between-study heterogeneity [54]. Despite its common use to assess heterogeneity, the I^2^ index only represent the percentage of variability in the effect sizes not caused by sampling error [55]. Therefore, as recommended, we additionally report PIs. The PIs are an equivalent of standard deviation and represent a range within which the effects of future studies are expected to fall based on current data [55,56]. 

To detect the presence of outliers that could potentially influence the estimated pooled effect and assess the robustness of our results, we applied an influence analysis based on the leave-one-out method [57]. If a study’s results had an important influence on the pooled effect, we conducted a sensitivity analysis, removing it or them. We additionally ran a drapery plot which is based on *p*-value functions and give us the *p*-value curve for the pooled estimate for all possible alpha levels [58].

To detect publication bias, we performed a visual evaluation of the Doi plot and the funnel plot [59], seeking asymmetry. We also performed a quantitative measure of the Luis Furuya-Kanamori (LFK) index, which has been shown to be more sensitive than the Egger test in detecting publication bias in a meta-analysis of a low number of studies [60]. An LFK index within ±1 represents no asymmetry, exceeding ±1 but within ±2 represents minor asymmetry, and exceeding ±2 involves major asymmetry. If there was significant asymmetry, we applied a small-study effect method to correct for publication bias using the Duval and Tweedie Trim and Fill Method [61]. 

## 3. Results

### 3.1. Characteristics of the Included Studies

A total of 58 RCTs were included [62,63,64,65,66,67,68,69,70,71,72,73,74,75,76,77,78,79,80,81,82,83,84,85,86,87,88,89,90,91,92,93,94,95,96,97,98,99,100,101,102,103,104,105,106,107,108,109,110,111,112,113,114,115,116,117]. We included a total of 8199 participants with a mean age ranging from 33.7 to 65.8 years. The patients were mostly women (N = 5764, 70.3%) diagnosed with chronic back pain [64,75,82,85,86,95,96], chronic low back pain [80,91,97,109,116,117], unspecific chronic pain [63,65,66,67,70,73,76,81,92,93,94,99,102,106,108,114,115,118,119], fibromyalgia [69,77,83,98,104,110,111,113], headache [79,100,101,107], rheumatic disorders [68,74,84,88,89,104,112], or others [71,72,78,87,90,105]. Details of the participant’s characteristics and studies are shown in Table 1. 

The studies compared online cognitive–behavioral therapy [64,65,66,67,68,69,73,75,85,90,91,94,95,96,99,104,116], acceptance and commitment therapy [76,81,92,98,102,119], self-management [83,93,99,103,106,108,109,111,113], mindfulness therapy [70,76,81,95,101,102,105], or other online behavioral techniques [62,63,71,72,74,77,78,79,80,82,84,86,87,88,89,97,100,107,110,112,114,115,117,118] against most frequently waiting list [65,66,69,70,74,79,81,85,90,92,95,96,100,102,103,108,110,112,113,116,118], usual care [68,73,75,77,82,83,84,86,91,93,94,98,99,101,104,106,107,109,111,117,119], or in-person intervention [67,70,76,78,88,89,104,109]. The intervention duration ranged between a single day [105] and 9 months [84]. The details of the interventions were described in Appendix C using the Behavior Change Technique Taxonomy (v1) [120].

### 3.2. Methodological Quality and Risk of Bias Results

The methodological quality of the studies was evaluated with the PEDro scale. The PEDro scores for each study are shown in Appendix D. In total, 36 were evaluated as having good quality [62,64,66,68,69,72,74,75,76,77,78,79,80,81,82,84,87,91,92,94,95,96,98,99,102,103,104,105,107,109,110,111,113,115,117,119] and 22 as having fair methodological quality [63,65,67,70,71,73,83,85,86,88,89,90,93,97,100,101,106,108,112,114,116,118]. The inter-rater reliability of the methodological quality assessment between assessors was high (κ = 0.901). The risk of bias of randomized trials was evaluated with the RoB2 tool. All the studies were rated as having a high risk of bias (100%). The risk of bias summary is shown in Appendix E. The inter-rater reliability of the risk of bias assessment between assessors was high (κ = 0.792).

### 3.3. Meta-Analysis Results

The overall strength of evidence for each variable and the reason it was downgraded is detailed in Table 2.

#### 3.3.1. Pain Intensity (vs. Usual Care/Waiting List)

**Figure 1:**
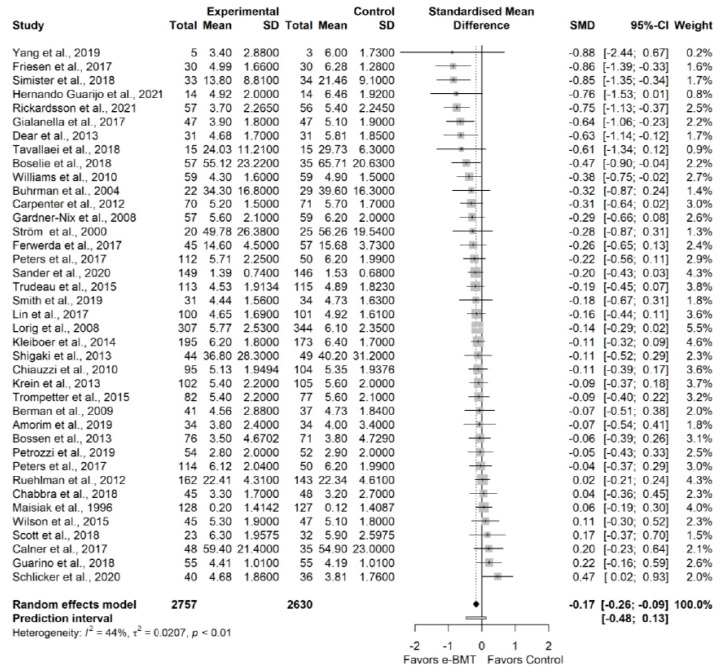
Sensitivity analysis of the pain intensity variable for online behavioral techniques against usual care or waiting list. The forest plot summarizes the results of included studies (sample size, mean, standard deviation (SD), standardized mean differences (SMDs), and weight). The small boxes with the squares represent the point estimate of the effect size and sample size. The lines on either side of the box represent a 95% confidence interval (CI).

The influence analyses revealed that the study from Hedman-Lagerlof et al. and Dear et al. were outliers [66,110], so, we ran a sensitivity analysis without them (Appendix F). The sensitivity analysis showed a statistically significant trivial effect size (38 RCTs; n = 5337; SMD = −0.17; 95% CI −0.26, −0.09) of e-BMT on pain intensity, with a significant heterogeneity (Q = 67.4 (*p* < 0.01), I^2^ = 44% (18%, 62%), PI −0.48, 0.13) and a low strength of evidence (Figure 1). Since PI crosses zero, we cannot be confident that future studies will not find contradictory results. The drapery plot revealed that the statistically significance of the results is robust through different *p*-value functions (Appendix F). With respect to the presence of publication bias, the visual evaluation of the shape of the funnel and Doi plot shown an asymmetrical pattern, showing a minor asymmetry (LFK index = −1.79) (Appendix F). When the sensitivity analysis is adjusted for publication bias, there is not anymore statistically significant effect (Appendix F). Subgroup analyses are detailed in Table 3.

#### 3.3.2. Pain Intensity (vs. In-Person BMT)

**Figure 2:**
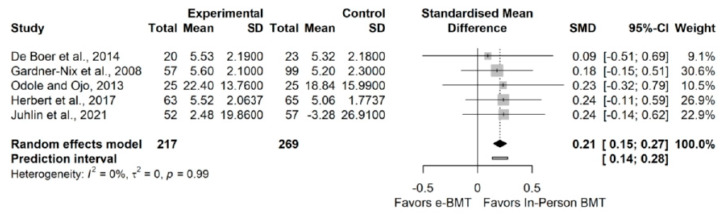
Synthesis forest plot of pain intensity variable of online behavioral techniques against in-person behavioral techniques. The forest plot summarizes the results of included studies (sample size, mean, standard deviation (SD), standardized mean differences (SMDs), and weight). The small boxes with the squares represent the point estimate of the effect size and sample size. The lines on either side of the box represent a 95% confidence interval (CI).

The influence analyses revealed no presence of outliers (Appendix G). The statistical analysis showed a statistically significant small effect size (5 RCTs; n = 486; SMD = 0.21; 95% CI 0.15, 0.27) of in-person BMT on pain intensity, with no significant heterogeneity (Q = 0.23 (*p* < 0.99), I^2^ = 0% (0%, 79.2%), PI 0.14, 0.28)) and a moderate strength of evidence (Figure 2). Since PI does not cross zero, we can be confident that future studies will not find contradictory results. The drapery plot revealed that the statistically significance of the results is robust through different *p*-value functions (Appendix G). With respect to the presence of publication bias, the visual evaluation of the shape of the funnel and Doi plot shown an asymmetrical pattern, showing a major asymmetry (LFK index = −2.36) (Appendix G). However, the adjustment did not influence the results (Appendix G). When the sensitivity analysis is adjusted for publication bias, there is no influence of the results (Appendix G).

#### 3.3.3. Pain Interference (vs. Usual Care/Waiting List)

**Figure 3:**
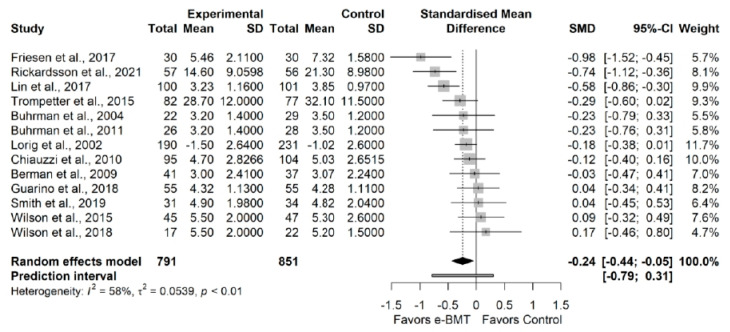
Synthesis forest plot of pain interference variable for online behavioral techniques against usual care or waiting list. The forest plot summarizes the results of included studies (sample size, mean, standard deviation (SD), standardized mean differences (SMDs), and weight). The small boxes with the squares represent the point estimate of the effect size and sample size. The lines on either side of the box represent a 95% confidence interval (CI).

The influence analyses revealed no presence of outliers (Appendix H). The statistical analysis showed a statistically significant small effect size (13 RCTs; n = 1642; SMD = −0.24; 95% CI −0.44, −0.05) of e-BMT on pain interference, with a significant heterogeneity (Q = 28.78 (*p* < 0.01), I^2^ = 58% (23%, 77%), PI −0.79, 0.31) and a low strength of evidence (Figure 3). Since PI crosses zero, we cannot be confident that future studies will not find contradictory results. We cannot be confident of the significance of our results, the drapery plot revealed that the statistically significance of the results did not maintain at *p* = 0.01 (Appendix H). With respect to the presence of publication bias, the visual evaluation of the shape of the funnel and Doi plot showed a symmetrical pattern, showing no asymmetry (LFK index = −0.21) (Appendix H). Subgroup analyses are detailed in Table 3.

#### 3.3.4. Kinesiophobia (vs. Usual Care/Waiting List)

**Figure 4:**
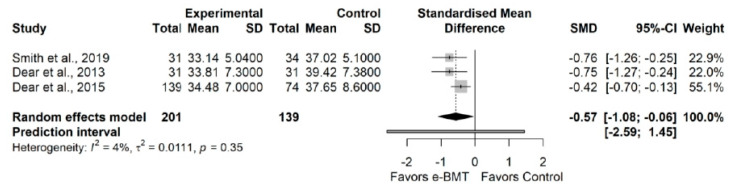
Sensitivity analysis of the kinesiophobia variable for online behavioral techniques against usual care or waiting list. The forest plot summarizes the results of included studies (sample size, mean, standard deviation (SD), standardized mean differences (SMDs), and weight). The small boxes with the squares represent the point estimate of the effect size and sample size. The lines on either side of the box represent a 95% confidence interval (CI).

The influence analyses revealed that the study from Friesen et al. was an outlier [69], so, we ran a sensitivity analysis without it (Appendix I). The sensitivity analysis showed a statistically significant small effect size (3 RCTs; n = 340; SMD = −0.57; 95% CI −1.08, −0.06) of e-BMT on kinesiophobia, with no significant heterogeneity (Q = 2.09 (*p* = 0.35), I^2^ = 4% (0%, 90%)) and a moderate strength of evidence (Figure 4). All the subscales of the pain catastrophizing scale were significantly improved. The drapery plot revealed that the statistically significance of the results is robust through different *p*-value functions (Appendix I). With respect to the presence of publication bias, the visual evaluation of the shape of the funnel and Doi plot showed an asymmetrical pattern, showing a major asymmetry (LFK index = −4.12) (Appendix G). When the sensitivity analysis was adjusted for publication bias, there still was a statistically significant small effect (Appendix I). 

#### 3.3.5. Catastrophizing (vs. Usual Care/Waiting List)

**Figure 5:**
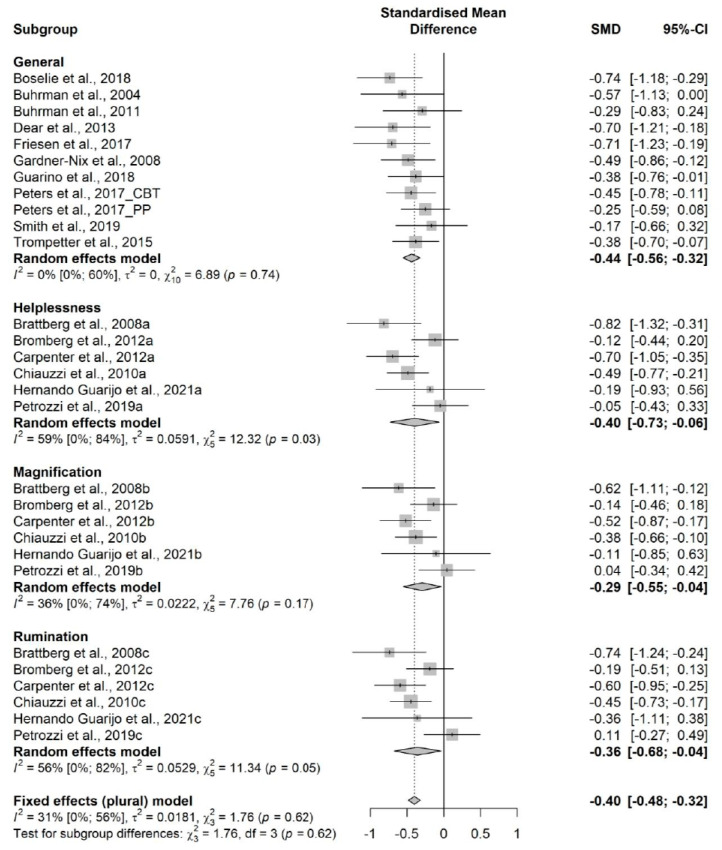
Sensitivity analysis of the catastrophizing variable and the subscales of the pain catastrophizing scale (Helplessness, Magnification and Rumination) for online behavioral techniques against usual care or waiting list. The forest plot summarizes the results of included studies (sample size, mean, standard deviation (SD), standardized mean differences (SMDs), and weight). The small boxes with the squares represent the point estimate of the effect size and sample size. The lines on either side of the box represent a 95% confidence interval (CI).

The influence analyses revealed that the studies from Ruehlman et al. and Trudeau et al. were outliers [93,103], so, we ran a sensitivity analysis without them (Appendix J). The sensitivity analysis showed a statistically significant small effect size (16 RCTs; n = 1613; SMD = −0.40; 95% CI −0.48, −0.32) of e-BMT on catastrophizing, with no significant heterogeneity (Q = 1.76 (*p* = 0.62) I^2^ = 31% (0%,56%)) and a moderate strength of evidence (Figure 5). All the subscales of the pain catastrophizing scale showed statistically significant improvements. The drapery plot revealed that the statistically significance of the results is robust through different *p*-value functions (Appendix J). With respect to the presence of publication bias, the visual evaluation of the shape of the funnel and Doi plot showed a symmetrical pattern, showing no asymmetry (LFK index = −0.34) (Appendix J). 

#### 3.3.6. Self-Efficacy (vs. Usual Care/Waiting List)

**Figure 6:**
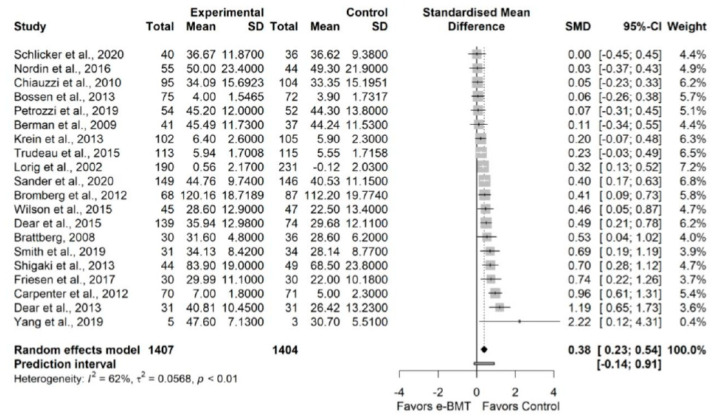
Sensitivity analysis of self-efficacy for online behavioral techniques against usual care or waiting list. The forest plot summarizes the results of included studies (sample size, mean, standard deviation (SD), standardized mean differences (SMDs), and weight). The small boxes with the squares represent the point estimate of the effect size and sample size. The lines on either side of the box represent a 95% confidence interval (CI).

The influence analyses revealed that the study from Kleiboer et al. was an outlier [79] (Appendix K) so, we ran a sensitivity analysis without it. The sensitivity analysis showed a statistically significant small effect size (20 RCTs; n = 2811; SMD = 0.38; 95% CI 0.17, 0.60) of e-BMT on self-efficacy, with a significant heterogeneity (Q = 50.41 (*p* < 0.01), I^2^ = 62% (29%, 80%), PI −0.14, 0.91) and a low strength of evidence (Figure 6). Since PI crosses zero, we cannot be confident that future studies will not find contradictory results. The drapery plot revealed that the statistically significance of the results is robust through different *p*-value functions (Appendix K). With respect to the presence of publication bias, the visual evaluation of the shape of the funnel and Doi plot showed a symmetrical pattern, showing a minor asymmetry (LFK index = 1.78) (Appendix K). When the sensitivity analysis was adjusted for publication bias, there was still a statistically significant small effect (Appendix K). Subgroup analyses are detailed in Table 3.

## 4. Discussion

The aim of this systematic review was to assess the effectiveness of e-BMT in pain-related variables in patients with musculoskeletal chronic pain. We found a trivial effect of e-BMT on pain intensity when compared with usual care or waiting list. Subgroup analyses showed that e-BMT seems to be more effective in fibromyalgia, internet-based or an application of more than 1 month. However, e-BMT showed a statistically significant lower improvement in pain intensity than an equivalent in-person BMT. There was a small effect on pain interference, kinesiophobia, and self-efficacy when compared with usual care or waiting list. Subgroup analyses showed that e-BMT seems to be more effective in unspecified chronic pain, CBT or self-management intervention, or an intervention that lasts between 1 and 3 months. There was a small effect on catastrophizing when compared with usual care or waiting list, however, when analyzed per item, all the subscales (helplessness, rumination and magnification and the overall score) showed a small effect in favor of e-BMT.

Dario et al. reviewed the effect of e-BMT on chronic LBP patients and found no effect on pain intensity [27]. We found that e-BMT had an overall significant effect on pain intensity, however, our subgroup analysis revealed no statistically significant effect for chronic LBP which confirms their results. Unlike us, they included only four studies in their meta-analysis. Du et al. reviewed the effect of online self-management on chronic LBP [24]. Unlike us, they found that an online e-BMT has similar effect in pain intensity, nonetheless, in the present systematic review we add a quantitative analysis to confirm that in-person BMT is more effective. We want to emphasize that there are no systematic reviews that provide meta-analyses on the effect of e-BMT, exclusively in adults, compared to usual care/waiting list on different important variables of the chronic pain patient (e.g., catastrophizing, pain interference, kinesiophobia, self-efficacy), nor that provide a quantitative comparison with in-person BMT.

The COVID-19 pandemic has confronted us with an important barrier to the appropriate management of the patient with chronic pain: social distancing [13,14]. Their treatments were undermined by this situation, resulting in a worsening of their condition [13,14]. Despite a current improvement of the COVID-19 pandemic situation, it has not concluded and the future is uncertain [121,122]. This leaves us with a question from which we must learn to prepare ourselves for the future: how to provide an effective rehabilitation to chronic pain patients when it is impossible to be physically present? TR and the use of new technologies appear as a serious answer to this problem and have been recommended worldwide [14,123]. Patients with chronic pain highlight the importance of health professionals to give them the tools to cope with the burden of chronic pain [124]. e-BMT offers the possibility to give to the patient tools to self-manage its condition through the different BMT (e.g., CBT, ACT) whatever the patient’s situation: from geographic isolation to social distancing. In the present systematic review, we found that e-BMT is effective in the management of the patient with chronic pain.

We found that in-person BMT was superior to e-BMT in improving pain intensity. Lewis et al. studied how patients perceived the transition from in-person to online treatment and found that 40% of patients thought the transition to online treatment may have affected the effectiveness of the treatment, and even more, 68% said they would not want to continue online when it would be possible to do so in person [125]. Our results could be explained by some patients’ preference for face-to-face treatment and, therefore, some patients may have the worst expectations about their treatment. Future studies should evaluate patient expectations of e-BMT as a possible confounding factor. Finally, the data must be considered with caution due to the heterogeneity of the sample, although a subgroup analysis was carried out to assess the effect of each intervention within BMTs and also within each specific clinical population. One of the things that the authors reflect on the results obtained is whether they are generalizable to all patients with persistent pain of musculoskeletal origin. The answer would be that it depends. First, it would have to be seen whether or not they have the presence of psychosocial variables such as catastrophic thoughts, movement-related fear or lack of self-efficacy. If these variables are not present, it would make little sense to implement interventions aimed at improving them. However, if they are present and can have an impact on the lives of patients with persistent pain, these tools should be considered. However, future studies are necessary, especially in order to homogenize the sample, something that is always sought after in the treatment of patients with pain.

### 4.1. Practical implication

About clinical implications, the results showed good results in favor of e-BMT. This gives us an effective treatment window in the COVID-19 era, so we are going to have a greater impact on patients with persistent pain. In addition, there is a decentralization of interventions, which may have some positive effects such as improving and increasing adherence to treatments due to easier accessibility, as well as lowering barriers to access or facilitating follow-up. Future studies should also focus on longer follow-ups to see this effectiveness and evaluate variables such as motivation or adherence to chronic pain treatments. Finally, telemedicine rehabilitation may lead to lower costs for both patients and therapists, which may reduce waiting lists for clinical treatments.

### 4.2. Limitations

Despite the use of subgroup analyses to study the heterogeneity between studies, the difference between the protocols of e-BMT prevents us to offer to health professionals a specific intervention design to implement. After adjusting for publication bias, our results on pain intensity versus usual care were no more statistically significant, so our results should be interpreted with caution. Our results on pain intensity, pain interference and self-efficacy are supported by only very low to low quality of evidence, true effects might be or are probably different from our estimated effects [126]. No study showed a low risk of bias according to the RoB2 scale, future studies should improve their quality to improve the confidence we can have in their results.

## 5. Conclusions

Based on the results obtained, e-BMT seems to be an effective option for the management of patients with musculoskeletal conditions with chronic musculoskeletal pain, especially in the era of COVID-19 where social distancing must be privileged. However, it does not appear superior to in-person BMT in terms of improving pain intensity.

## Figures and Tables

**Table 1:** Details of the studies included in the systematic review.

Authors, Year Design Country	Participants Sample Size (n) Age (Mean (SD)) Gender *Condition*	Intervention Modality *Format*	Comparator	Outcomes	Results
**Amorim et al., 2019** Pilot RCT Australia	N = 68 58.3 (13.4) yrs 50%F/50%M ***Chronic LBP***	Tailored-plan treatment with activity tracker and monitoring application. + Telephone follow-up ***Mobile application***	Advice to stay active and booklet about benefits of physical activity	- *Pain intensity: NRS (0–10)*	No significant differences in pain intensity.
**Berman et al., 2009** RCT USA	N = 89 65.8 (N/R) yrs 87%F/13%M ***Unspecified chronic pain***	Self-care intervention ***Internet-based***	No intervention	- *Pain intensity (average, worst, least): BPI* - *Pain interference: BPI* - *Self-efficacy: PSEQ*	Significant difference in pain intensity (Self-care: *p* < 0.01 and control: *p* < 0.05) and pain interference (both *p* < 0.01), but without differences between group. Small no-significant improvement in self-efficacy in both groups (*p* > 0.05).
**Boselie et al., 2018** RCT The Netherlands	N = 33 N/R yrs N/R %F/N/R %M ***Unspecified chronic pain***	Positive psychology ***Internet-based***	Waiting list	- *Pain intensity: VAS*	Intervention group effect was non-significant for pain intensity (*p* = 0.16).
**Bossen et al., 2013** RCT The Netherlands	N = 199 62.0 (5.7) yrs 65%F/35%M ***Knee and hip OA***	Behavior graded activity program ***Internet-based***	Waiting list	- *Pain intensity: NRS (0–10)* - *Self-Efficacy: ASES*	No significant differences in pain intensity and self-efficacy.
**Brattberg, 2008** RCT Sweden	N = 66 43.8 (8.8) yrs 100%F ***Unspecified chronic pain***	Emotional freedom techniques **Internet-based**	Waiting list	- *Catastrophizing: PCS* - *Self-efficacy: GSES*	Statistically significant time × group interaction in the different subscales of the pain catastrophizing scale (*p* < 0.001, *p* = 0.006 and *p* < 0.001). There was no statistically significant difference in self-efficacy.
**Bromberg et al., 2012** RCT USA	N = 189 42.6 (11.5) yrs 89%F/11%M ***Chronic migraine***	Structured behavior changes program +Usual care **Internet-based**	Usual care	- *Headache severity (1–4)* - *Self-efficacy: Headache Management Self-Efficacy Scale* - *Pain catastrophizing: PCS*	They also showed less feeling of helplessness (*p* = 0.003) and rumination (*p* = 0.0003), globally, there was a higher improvement of catastrophizing (*p* = 0.0006). There was also a higher improvement of self-efficacy (*p* < 0.0001).
**Buhrman et al., 2004** RCT Sweden	n = 56 44.6 (10.4) yrs 63%F/37%M ***Chronic back pain***	Online CBT + Relaxation with CDs + Telephone calls about goals **Internet-based**	Waiting list	- *Pain severity and Pain interference: MPI* - *Pain intensity: NRS (0–100) Average and Highest*	Significant effect of intervention group on catastrophizing (*p* < 0.01). There was no significant main effects difference on multidimensional pain inventory. Both groups reduced their average and highest pain intensity (*p* < 0.05) without significant differences.
**Buhrman et al., 2011** RCT Sweden	N = 54 43.2 (9.8) yrs 69%F/32%M ***Chronic back pain***	Online CBT **Internet-based**	Waiting list	- *Catastrophizing: CSQ Catastrophizing subscale* - *Pain interference: MPI*	There is a significant interaction for the intervention group (*p* = 0.0001) on catastrophizing. However, there were no significant differences between group for multidimensional pain inventory.
**Calner et al., 2017 & Nordin et al., 2016** RCT Sweden	N = 99 43.1 (10.5) yrs 85%F/15%M ***Unspecified chronic pain***	Multimodal pain rehabilitation + Behavior change program **Internet-based**	Multimodal pain rehabilitation	- *Pain intensity: VAS*	There were no statistically significant differences over time on pain intensity.
**Carpenter et al., 2012** RCT USA	N = 164 42.5 (10.3) yrs 83%F/17%M ***Chronic LBP***	Interactive self-help intervention (pain education and CBT) **Internet-based**	Waiting list	- *Pain catastrophizing: PCS* - *Self-Efficacy: ASES* - *Pain intensity: NRS (Average, highest, lower)*	Both groups improved significantly all the outcomes.
**Chabbra et al., 2018** RCT India	N = 93 41.2 (14.1) yrs N/R %F/N/R %M ***Chronic LBP***	Daily activity goals with exercises **Mobile application**	Prescription about medicines and advice about physical activity	- *Pain intensity: NRS*	Both groups showed a significant decrease of pain intensity (*p* < 0.001) but without differences.
**Chiauzzi et al., 2010** RCT USA	N = 209 46.1 (12.0) yrs 68%F/32%M ***Chronic back pain***	Online CBT and self-management website **Internet-based**	Standard back pain management text materials	- *Pain intensity: BPI* - *Catastrophizing: PCS* - *Self-efficacy: PSEQ*	There was no statistically significant effect on self-efficacy, pain intensity, and pain catastrophizing.
**Choi et al., 2019** RCT	N = 84 54.5 (x) yrs 68%F/32%M ***Frozen shoulder***	NSAIDs + Self-Exercise+ mobile-based guided exercise **Mobile application**	NSAIDs + Exercise	*Pain intensity*: *VAS*	There were no significant differences between groups in any outcomes.
**De Boer et al., 2014** RCT The Netherlands	N = 50 52.1 (11.2) yrs 64%F/36%M ***Unspecified chronic pain***	CBT **Internet-based**	CBT Face-to-Face	- *Pain catastrophizing: PCS* - *Pain intensity: VAS (0–10)* - *Pain interference: VAS (0–10)*	Online group showed a statistically significant interaction on catastrophizing (*p* = 0.023), pain intensity (*p* = 0.020), however there was no interaction in other outcomes.
**Dear et al., 2013** RCT Australia	N = 63 49.0 (13) yrs 85%F/15%M ***Unspecified chronic pain***	Online CBT **Internet-based**	Waiting list	- *Duration, severity, location, and level of interference of pain: WBPQ* - *Self-efficacy: PSEQ* - *Kinesiophobia: TSK-17* - *Catastrophizing: PRSS*	Intervention had a significantly higher post-treatment improvement self-efficacy (*p* < 0.001), kinesiophobia (*p* < 0.001) and the catastrophizing subscale of the PRSS (*p* = 0.005).
**Dear et al., 2015** RCT Australia	N = 490 50 (13) yrs 80%F/20%M ***Unspecified chronic pain***	G1: Online CBT + Regular online contact G2: Online CBT + optimal online contact G3: Online CBT **Internet-based**	Waiting list	- *Location, severity and duration of pain: WBPQ* - *Self-efficacy: PSEQ* - *Kinesiophobia: TSK-17*	Intervention groups had significantly a significantly lower scores of pain intensity average than waiting list (*p* ≤ 0.03). All treatment groups, without control group, showed a significant improvement of self-efficacy and kinesiophobia (*p* ≤ 0.046).
**Ferwerda et al., 2017** RCT The Netherlands	N = 133 56.4 (10) yrs 64%F/36%M ***Rheumatoid arthritis***	CBT **Internet-based**	Usual care	- *Pain intensity: Pain subscale of the IRGL*	There was no statistically significant improvement of pain intensity (*p* = 0.35).
**Friesen et al., 2017** RCT Canada	N = 60 48.0 (11.0) yrs 95%F/5%M ***Fibromyalgia***	CBT + Telephone calls **Internet-based**	Waiting list	- *Pain intensity and interference: BPI* - *Self-efficacy: PSEQ* - *Pain-related cognitions: Catastrophizing and coping subscales of PRSS* - *Kinesiophobia: TSK-17*	Intervention group had a significantly higher improvement of pain intensity (*p* = 0.037). However, there was not for pain interference. There was also a statistically significant time by group interaction for kinesiophobia (*p* < 0.001). Other outcomes were not significant.
**Gardner-Nix et al., 2008** RCT Canada	N = 163 50.0–55.0 yrs 81%F/19%M ***Unspecific chronic pain***	Mindfulness **Videoconferencing**	CG1:Mindfulness Face-to-Face CG2: Waiting list	- *Catastrophizing: PCS* - *Pain intensity: NRS*	Both mindfulness group improved more catastrophizing than waiting list (*p* < 0.01) post-treatment but without significant differences between them. Both mindfulness group showed lower pain-intensity than control group post-treatment (*p* < 0.01 and *p* < 0.05), but face-to-face showed also lower pain score than online treatment (*p* < 0.05).
**Gialanella et al., 2017 and 2020** RCT Italy	N = 94 58.1 (12.7) yrs 89%F/11%M ***Chronic neck pain***	Exercise + Telephone calls with a therapist **Telephone**	Exercise + Recommendations to continue to exercise	- *Pain intensity: VAS*	Both groups had statistically significant lower pain intensity post-treatment (*p* < 0.001), but it was lower in the intervention group (*p* < 0.001).
**Guarino et al., 2018** RCT USA	N = 110 51.3 (10.9) yrs 60%F/40%M ***Unspecific chronic pain***	Online CBT + Usual care **Internet-based**	Usual care	- *Pain severity and pain interference: MPI* - *Catastrophizing: PCS*	Both groups significantly improved pain severity and interference, but without difference between them. However, patients with the online treatment showed a statistically significant reduction catastrophizing (*p* = 0.040) in comparation with control group.
**Heapy et al., 2017** RCT USA	N = 125 57.9 (11.6) yrs 22%F/78%M ***Chronic back pain***	CBT **Interactive voice response**	Face-to-Face CBT	- *Pain intensity: NRS (0–10)* - *Pain interference: Interference subscale of WHYMPI*	CBT through interactive voice response was noninferior to in-person CBT in post-treatment pain intensity. There were no significant differences between e-CBT and face-to-face CBT in pain interference.
**Hedman-Lagerlöf et al., 2018** RCT Sweden	N = 140 98%F/2%M 50.8 (24–77) yrs ***Fibromyalgia***	Online exposure therapy **Internet-based**	Waiting list	- *Pain intensity: FIQ*	There were statistically significant interactions in favor of intervention group on pain intensity according to the FIQ, (*p* < 0.001).
**Herbert et al., 2017** RCT USA	N = 128 18%F/82%M 52.0 (13.3) yrs ***Unspecific chronic pain***	ACT **Video teleconferencing**	Face-to-face ACT	- *Pain interference: BPI* - *WHMPI*	VTC-ACT was noninferior to face-to-face ACT on pain interference. Also, there were no significant differences on any other outcomes, except on the activity subscale of the MPI (*p* = 0.03).
**Hernando-Garijo et al., 2021** RCT Spain	N = 34 53.4 (8.8) yrs 100%F ***Fibromyalgia***	Video-guided aerobic training + usual medical prescription **Videos**	Usual medical prescription	- *Pain intensity: VAS* - *Catastrophizing: PCS*	There was a statistically significant higher improvement of pain intensity (*p* = 0.021). There was no statistically significant difference in catastrophizing.
**Juhlin et al., 2021** RCT Sweden	N = 139 47.6 (10.1) yrs 90%F/10%M ***Chronic widespread pain***	Person-centered intervention supported by online platform **Internet-based**	Person-centered intervention	- *Pain intensity: Pain subscale of the FIQ* - *Self-efficacy: GSES*	There were no significant differences between group on pain intensity (*p* = 0.39) or other outcomes.
**Kleiboer et al., 2014** RCT The Netherlands	N = 368 43.6 (11.5) yrs 85%F/15%M ***Migraine***	Online behavioral training **Internet-based**	Waiting list	- *Attack peak intensity* - *Self-efficacy: HMSE*	There were no significant differences between groups except for self-efficacy (*p* < 0.001).
**Krein et al., 2013** RCT USA	N = 229 51.6 (12.6) yrs 12%F/88%M ***Chronic LBP***	Pedometer, online goal-setting and feedback platform and e-community **Internet-based**	Pedometer	- *Pain interference: MOS* - *Pain intensity: NRS (0–10)* - *Self-efficacy for exercise: Exercise Self-efficacy score*	Intervention group showed no statistically significant on pain interference (*p* = 0.09). Intervention group showed a higher exercise self-efficacy post-treatment (*p* = 0.01) who failed to maintain at 12 months. There were no more significant differences.
**Lin et al., 2017** RCT Germany	N = 201 51.0 (12.4) yrs 86%F/14%M ***Unspecific chronic pain***	Online guided ACT **Internet-based**	Waiting list	- *Pain interference: MPI* - *Pain intensity: NRS*	There was a significant interaction effect for group x time on the pain interference (*p* < 0.01), but also on pain intensity (*p* < 0.05), in favor of intervention group.
**Lorig et al., 2002** RCT USA	N = 580 45.5 (N/R) yrs 38%F/62%M ***Chronic back pain***	Back pain textbook via e-mail + videotapes about back pain experiences + e-community **Online textbook and videotapes and internet-based**	Usual care + subscription to a non-health-related magazine	- *Pain interference: VAS* - *Self-efficacy: N/R*	There was a statistically significant higher improvement in pain intensity (*p* < 0.05) in intervention group. There was also a significant higher improvement of self-efficacy (*p* = 0.003).
**Lorig et al., 2008** RCT USA	N = 855 52.3 (11.6) yrs 90%F/10%M ***Fibromyalgia***	Web-based self-management instruction and discussion **Internet-based**	Usual care	- *Pain intensity: VAS*	There was a significant time by group interaction on pain intensity (*p* < 0.001).
**Maisiak et al., 1996** RCT USA	N = 255 60.3 (N/R) yrs 92%F/8%M ***Hip or Knee OA or Rheumatoid Arthritis***	Telephone counseling strategy **Telephone**	Usual care	- *Physical aspect, pain scores and affect: AIMS2*	Patients in the telephone counselling had higher improvement in total AIMS2 score (*p* < 0.01).
**Moessner et al., 2012** RCT Germany	N = 75 45.9 (9.1) yrs 56%F/44%M ***Chronic back pain***	Self-monitoring + Online guided chat **Internet-based**	Usual care	- *Pain intensity: NRS (0–10) and SF-36 Pain subscale*	Patients had a statistically significant lower score of pain according to the SF536 Pain subscale. However, there were no differences in other outcomes.
**Odole and Ojo, 2013 and 2014** RCT Nigeria	N = 50 55.5 (7.6) yrs 48%F/52%M ***Knee OA***	Phone-based Physical Therapy **Telephone**	Face-to-face physical therapy	- *Pain intensity: VAS (0–100)*	Both groups showed statistically significant improvement of their pain intensity.
**Peters et al., 2017** RCT Sweden	N = 284 48.6 (12.0) yrs 85%F/15%M ***Chronic back, neck or shoulder pain***	G1: Online Positive psychology G2: Online CBT **Internet-based**	Waiting list	- *Pain intensity: NRS (0–10)* - *Catastrophizing: PCS*	There were significant differences in pain catastrophizing and helplessness. There was no statistically significant time, group, or time by group effect on pain intensity.
**Petrozzi et al., 2019** RCT New Zealand	N = 108 50.4 (13.6) yrs 50%F/50%M ***Chronic LBP***	Online CBT+ Usual care **Internet-based**	Usual care	- *Self-efficacy: PSEQ* - *Catastrophizing: PCS* - *Pain intensity: NRS*	There were no statistically significant differences between the two groups on self-efficacy (*p* = 0.52), pain intensity (*p* = 0.95) and catastrophizing (*p* = 0.89) at any time-points.
**Rickardsson et al., 2021** RCT Sweden	N = 113 49.5 (12.1) yrs 75%F/25%M ***Unspecific chronic pain***	Online ACT **Internet-based**	Waiting list	- *Pain interference: PII* - *Pain intensity: NRS*	The intervention group showed significant interaction effects of time x group for pain interference (*p* < 0.001) and pain intensity (*p* = 0.004).
**Ruehlman et al., 2012** RCT USA	N = 305 44.9 (x) yrs 64%F/36%M ***Unspecific chronic pain***	Online program about chronic pain with self-management tools and a e-community **Internet-based**	Usual care	- *Pain severity, pain interference and emotional burden: PCP-S* - *Prior diagnoses, pain characteristics, pain location, medication use and health care status, coping, catastrophizing, attitudes and belief, social responses: PCP-EA*	Intervention group showed a significant group × time interaction in pain interference (*p* = 0.00) and pain severity (*p* = 0.01). Intervention group also showed a significant group × time interaction in catastrophizing (*p* = 0.01)
**Sander et al., 2020** RCT Germany	N = 295 52.8 (7.7) yrs 62%F/38%M ***Unspecific chronic pain***	Online CBT + Usual care **Internet-based**	Usual Care	- *Pain intensity: NRS* - *Self-efficacy: PSEQ*	Online training showed small to medium effect sizes in all the outcomes, except for pain intensity.
**Schlickler et al., 2020** RCT Germany	N = 76 50.8 (7.9) yrs 55%F/45%M ***Chronic back pain***	Online CBT-based intervention **Internet-based and mobile-based**	Waiting list	- *Pain intensity: NRS (worst, least and average)* - *Self-efficacy: PSEQ*	There were no statistically significant differences in any other outcome.
**Schulz et al., 2007** RCT Switzerland	N = 35 45.3 (N/R) yrs 29%F/71%M ***Chronic low back pain***	Online social and educational about pain management website **Internet-based**	No treatment	- *Pain intensity: NRS*	Pain intensity in the treatment group has decreased, however, there was no change in the control group.
**Shigaki et al., 2013** RCT USA	N = 108 49.8 (11.9) yrs 94%F/6%M ***Rheumatoid arthritis***	Education and social network website + Telephone calls **Internet-based**	Waiting list	- *Pain intensity: RADAR* - *Self-efficacy: ASES*	There were significant differences post-treatment in favor of the intervention group in self-efficacy (*p* = 0.000) and quality of life (*p* = 0.003), who maintained at 9 months (*p* = 0.000 and *p* = 0.004, respectively).
**Scott et al., 2018** RCT UK	N = 63 45.5 (14.0) yrs 64%F/36%M ***Unspecific chronic pain***	Online ACT + Usual care **Internet-based**	Usual care	- *Pain interference: BPI* - *Pain intensity and pain distress: NRS*	Pain interference and pain intensity showed small effect size in favor of intervention group.
**Simister al., 2018** RCT	N = 67 39.7 (9.4) yrs 95%F/5%M ***Fibromyalgia***	Online ACT + Usual care **Internet-based**	Usual care	- *Pain intensity: SF-MPQ* - *Kinesiophobia: TSK-11* - *Catastrophizing: PCS*	Intervention group significantly improved, relative to control group, kinesiophobia (*p* < 0.001). Small effect size for pain in favor of intervention group (0.11). There was only a tendency to improvement in favor of online group on catastrophizing (*p* = 0.051).
**Smith et al., 2019** RCT Australia	N = 80 45.0 (13.9) yrs 88%F/12%M ***Unspecific chronic pain***	Online self-management and CBT-based intervention **Internet-based**	Usual care	- *Self-efficacy: PSEQ* - *Pain severity and pain interference: BPI* - *Catastrophizing: PCS* - *Kinesiophobia: TSK*	There were significant time-by-group interactions on pain self-efficacy (*p* < 0.05), pain severity (*p* < 0.05), kinesiophobia (*p* < 0.01), in favor of intervention group. However, there were no interactions for pain interference.
**Ström et al., 2000** RCT Sweden	N = 45 36.7 (N/R) yrs 69%F/31%M ***Recurrent headache sufferers***	Online relaxation and problem-solving intervention **Internet-based**	Wait-list	- *Pain intensity: NRS (0–100)*	There was a statistically significant difference between groups at post treatment for pain intensity (*p* = 0.009).
**Tavallaei et al., 2018** RCT Iran	N = 30 33.7 (9.0) yrs 100%F ***Migraine and tension-type headache***	Mindfulness-based Stress Reduction Bibliotherapy **Internet-based**	Usual care	- *Pain intensity: SF-MPQ*	There was a significant difference between both groups in favor of the online group in pain intensity (*p* = 0.035).
**Trompetter et al., 2015** RCT The Netherlands	N = 238 52.7 (12.4) yrs 76%F/24%M ***Unspecific chronic pain***	Online ACT **Internet-based**	Waiting list	- *Pain interference: MPI* - *Catastrophizing: PCS*	There was no significant difference in pain interference, however there was in pain intensity (*p* = 0.35) and catastrophizing (*p* = 0.019).
**Trudeau et al., 2015** RCT USA	N = 228 49.9 (11.6) 68%F/32%M ***Arthritis***	Online self-management intervention **Internet-based**	Waiting List	- *Self-efficacy: ASES* - *Catastrophizing: PCS* - *Pain severity and pain interference: BPI-SF*	There were statistically significant interactions group-by-time in favor of intervention group on self-efficacy (*p* = 0.0293) and catastrophizing (*p* = 0.0055).
**Vallejo et al., 2015** RCT Spain	N = 60 51.6 (9.9) yrs 100%F ***Fibromyalgia***	Online CBT + Usual care **Internet-based**	G1: Face-to-face CBT + Usual care G2: Usual care	- *Catastrophizing: PCS* - *Self-efficacy: CPSES*	Both CBT groups showed improvement in catastrophizing (both, *p* < 0.001). Only the online group showed improvement of self-efficacy (*p* < 0.001).
**Westenberg et al., 2018** RCT USA	N = 126 54.5 (15.0) yrs 50%F/50%M	Online Mindfulness	Attention control	- *Pain intensity: NRS*	Online Mindfulness showed a statistically significant higher improvement of pain intensity (*p* = 0.008). However, the difference in pain intensity did not reach the minimal clinically important difference.
**Williams et al., 2010** RCT USA	N = 118 50.5 (11.5) yrs 95%F/5%M ***Fibromyalgia***	Online self-management + Usual care **Internet-based**	Usual care	- *Pain intensity: BPI*	Patients in the intervention group shown statistically significant improvement of pain intensity (*p* < 0.01).
**Wilson et al., 2015** RCT USA	N = 114 49.3 (11.6) yrs 78%F/12%M ***Unspecific chronic pain***	Online pain self-management program **Internet-based**	Usual care	- *Pain severity and pain interference: BPI* - *Self-efficacy: PSEQ*	There was not a statistically significant interaction group by time on pain interference and pain intensity. However, there was a significant interaction group by time on self-efficacy (*p* = 0.00) in favor of the online group.
**Wilson et al., 2018** RCT USA	N = 60 44.3 (12.0) yrs 44%F/56%M ***Unspecific chronic pain***	Online self-management program **Internet-based**	Waiting-list	- *Self-efficacy: PSEQ* - *Pain severity and pain interference: BPI*	Intervention group showed higher level of pain interference, and pain severity, than control group.
**Yang et al., 2019** RCT China	N = 8 40.8 (12.5) yrs 88%F/12%M ***Chronic LBP***	Online self-management + Face-to-face Physiotherapy ***Mobile application***	Face-to-face physiotherapy	- *Current pain intensity: VAS (0–100)* - *Self-efficacy: PSEQ*	There were no significant differences on pain intensity. Additionally, there were no significant interaction effects on self-efficacy.

Abbreviatures: %F: Women proportion; %M: Men proportion; ACT: Acceptance and Commitment therapy; AIMS2: Arthritis Impact Measurement Scales-2; ASES: Arthritis Self-Efficacy Scale; BPI: Brief Pain Inventory-Short form; CBT: Cognitive–behavioral therapy; CG: Control group; CPCI: Chronic Pain Coping Inventory; CPSES: Chronic Pain Self-efficacy Scale; FIQ: Fibromyalgia Impact Questionnaire; GSES: General Self-Efficacy Scale; HMSE: Headache Management Self-Efficacy questionnaire; IRGL: Impact of Rheumatic Diseases on General Health and Lifestyle; KOOS: Knee Osteoarthritis Outcome Score; LBP: Low back pain; MOS: Medical Outcomes Study; MPI: Multidimensional pain inventory; NRS: Numeric rating scale; NSAIDs: nonsteroidal anti-inflammatory drugs; PCS: Pain Catastrophizing Scale; PCP-EA: Profile of Chronic Pain Extended Assessment; PCP-S: Profile of Chronic Pain: Screen; PII: Pain Interference Index; PSEQ: Pain Self-efficacy Questionnaire; PRSS: Pain Responses Self-Statements; RADAR: Rapid Assessment of Disease Activity in Rheumatology; SF-36: 36-Item Short Form Health Survey questionnaire; SF-MPQ: Short Form McGill Pain Questionnaire; TSK: Tampa Scale of Kinesiophobia; VAS: Visual analogue scale; VTC: Video-teleconferencing; WHMPI: West Haven–Yale Multidimensional Pain Inventory; WPBQ: Wisconsin Brief Pain Questionnaire.

**Table 2:** Summary of findings and quality of evidence (GRADE).

Certainty Assessment							No. of Participants		Effect	Certainty
Outcome (No. of Studies)	Study Design	Risk of Bias	Inconsistency	Indirectness	Imprecision	Publication Bias	e-BMT	Control	Absolute (95% CI)	
*Pain intensity* *(vs. Usual care/Waiting list) (n* = *38)*	RCT	Serious	Not serious	Not serious	Not serious	Serious	2757	2580	−0.17 (−0.26; −0.09)	**Low** **⊕⊕**
*Pain intensity* *(vs. In person BMT)* *(n = 5)*	RCT	Serious	Not serious	Not serious	Not serious	Not serious	217	269	0.21 (0.15; 0.27)	**Moderate** **⊕⊕⊕**
*Pain interference* *(vs. Usual care/Waiting list) (n = 13)*	RCT	Serious	Serious	Not serious	Not serious	Not serious	791	851	−0.24 (−0.44; −0.05)	**Low** **⊕⊕**
*Kinesiophobia* *(vs. Usual care/Waiting list) (n = 3)*	RCT	Serious	Not serious	Not serious	Not serious	Not serious	201	139	−0.57 (−1.08; −0.06)	**Moderate** **⊕⊕⊕**
*Catastrophizing* *(vs. Usual care/Waiting list) (n = 16)*	RCT	Serious	Not serious	Not serious	Not serious	Not serious	826	787	−0.40 (−0.48; −0.32)	**Moderate** **⊕⊕⊕**
*Self-efficacy* *(vs. Usual care/Waiting list) (n = 20)*	RCT	Serious	Serious	Not serious	Not serious	Not serious	1407	1404	0.38 (0.23; 0.54)	**Low** **⊕⊕**

CI: Confidence interval, e-BMT: Online Behavioral Modification Techniques, RCT: Randomized controlled trial.

**Table 3:** Subgroup analyses of the pain intensity, pain interference and self-efficacy outcomes.

Outcomes (Contrast)—*Subanalysis*	N Studies	SMD	Lower Limit 95%CI	Upper Limit 95% CI	Q	I^2^
***Pain intensity (vs. Usual Care/Waiting List)***—*Treatment*						
*ACT*	5	−0.33	−0.86	0.19	15.40	74%
*CBT*	12	−0.18	−0.38	0.02	23.16	53%
*Positive Psychology*	2	−0.23	−2.96	2.50	2.45	59%
*Self-management*	8	−0.11	−0.23	0.008	6.48	0%
*Mindfulness*	2	−0.35	−1.97	1.26	0.58	0%
*Other types of treatment*	10	−0.11	−0.27	0.05	15.40	74%
***Pain intensity (vs. Usual Care/Waiting List)*** —*Chronic Musculoskeletal disorder*						
*Unspecific back pain*	6	−0.16	−0.50	0.19	13.21	62%
*Fibromyalgia*	4	−0.66	−1.06	−0.25	3.28	9%
*Headache*	3	−0.16	−0.55	0.23	1.79	0%
*Low Back Pain*	6	−0.12	−0.28	0.04	3.34	0%
*Rheumatic disorders*	5	−0.09	−0.25	0.07	2.74	0%
*Unspecified chronic pain*	15	−0.14	−0.29	0.01	27.33	49%
***Pain intensity (vs. Usual Care/Waiting List)***—*Online Modality*						
*Mobile application*	3	−0.04	−0.57	0.50	1.31	0%
*Internet*	30	−0.18	−0.26	−0.10	44.29	35%
*Multi-device*	2	0.33	−1.40	2.07	0.72	0%
*Videoconference*	2	−0.40	−2.92	2.13	1.17	15%
*Telephone*	2	−0.27	−4.71	4.16	8.08	88%
***Pain intensity (vs. Usual Care/Waiting List)***—*Intervention duration (without Krein et al.)*						
*More than 3 months*	11	−0.16	−0.32	−0.002	16.60	40%
*Between 1 and 3 months*	24	−0.18	−0.32	−0.05	48.79	53%
*Less than 1 month*	3	−0.21	−0.61	0.20	1.54	0%
***Pain interference (vs. Usual Care/Waiting List)***—*Treatment*						
*ACT*	3	−0.52	−1.07	0.03	3.53	43%
*CBT*	6	−0.22	−0.59	0.16	10.89	54%
*Self-Management*	4	−0.09	−0.32	0.14	2.29	0%
***Self-efficacy (vs. Usual Care/Waiting List)***—*Treatment*						
*CBT*	9	0.49	0.17	0.80	33.21	76%
*Self-management*	6	0.32	0.13	0.50	5.65	12%
*Other types of treatment*	5	0.27	−0.06	0.59	8.06	50%
***Self-efficacy (vs. Usual Care/Waiting List)***—*Chronic Musculoskeletal disorder*						
*Unspecific back pain*	4	0.24	−0.06	0.54	5.37	44%
*Fibromyalgia*	2	0.63	−0.72	1.97	0.33	0%
*LBP*	4	0.52	−0.54	1.58	17.75	83%
*Headache*	1	0.41	0.09	0.73	N/A	N/A
*Rheumatic disorders*	4	0.24	−0.22	0.70	6.93	57%
*Unspecified chronic pain*	5	0.56	0.09	1.02	9.75	59%
***Self-efficacy (vs. Usual Care/Waiting List)***—*Intervention duration (without Krein et al.)*						
*More than 3 months*	3	0.37	−0.13	0.87	2.72	27%
*Between 1 and 3 months*	13	0.37	0.17	0.56	27.17	56%
*Less than 1 month*	3	0.74	−1.49	2.97	18.97	90%

Abbreviatures: ACT: Acceptance and Commitment therapy; CBT: Cognitive–behavioral therapy; CI: Confidence interval; LBP: low back pain; N/A: Not Applicable; SMD: Standardized mean differences.

## Data Availability

Not applicable.

## Appendix B. Search Strategies in the Different Electronic Databases

**Pubmed—350 results**

((“Web”) OR (“ehealth”) OR (“mhealth”) OR (“remote treatment”) OR (“digital treatment”) OR (“Mobile Applications”[MesH]) OR (“Software”[Mesh]) OR (“Online”) OR (“Telephone”) OR (“Cell phone”[MesH]) OR (“eTherapy”) OR (“Internet”) OR (“Online”) OR (“Telerehabilitation”) OR (“Internet-Based Intervention”[MesH]) OR (“Telerehabilitation”[MesH]) OR (Telemedicine[MesH])) AND ((“Chronic Pain”) OR (“Chronic Pain”[Mesh])) AND (randomized controlled trial[pt] OR controlled clinical trial[pt] OR randomized[tiab] OR placebo[tiab] OR clinical trials as topic[mesh:noexp] OR randomly[tiab] OR trial[ti] NOT (animals[mh] NOT humans [mh]) NOT (“protocol”) NOT (“Review”))

**CINAHL—173 results**

(web or internet or online or mobile or remote treatment or digital treatment or Internet-Based Intervention or Telerehabilitation or Telemedicine) AND (chronic pain or persistent pain or long term pain or long-term pain) AND (randomized controlled trials or rct or randomised control trials) NOT (systematic review or meta-analysis or literature review or review of literature) NOT (pediatric or child or children or infant or adolescent)

**Psychology and Behavioral Sciences Collection (EBSCO)—12 results**

(web or internet or online or mobile or remote treatment or digital treatment or Internet-Based Intervention or Telerehabilitation or Telemedicine or) AND (chronic pain or persistent pain or long term pain or long-term pain) AND (randomized controlled trials or rct or randomised control trials) NOT (systematic review or meta-analysis or literature review or review of literature) NOT (pediatric)

**APA PsychINFO—75 results**

(web or websites or internet or online or Online Therapy or mobile or Mobile Applications or remote treatment or digital treatment or Digital Interventions or Internet-Based Intervention or Telerehabilitation or Telemedicine) AND (chronic pain or persistent pain or long term pain or long-term pain) AND (randomized controlled trials or rct or randomised control trials) NOT (systematic review or meta-analysis or literature review or review of literature) NOT (pediatric or child or children or infant or adolescent)

**Web of science—49 studies**

TI = (Web OR eearth OR melth OR remote treatment OR digital treatment OR Mobile Applications OR Software OR Online OR Telephone OR Cell phone OR estherapy OR Internet OR Online OR Telerehabilitation OR Internet-Based Intervention OR Telerehabilitation OR Telemedicine) AND TI = (Chronic pain) AND TI = (randomi?ed controlled trial* OR rct)

**Google Scholar **

(“web” OR “online” OR “internet” OR “mobile” OR “telerehabilitation” OR “telemedicine”) AND [allintitle:”chronic pain” OR “persistent pain”] AND (“randomized controlled trial” OR “randomised controlled trial OR “RCT”)-review

## Appendix C. Details of the Interventions

**Table:**

Authors, Year	Intervention			Comparator		
	Format Equipment and Contact Form	Modality and Content	Duration and Frequency, Follow-Up	Format Equipment	Modality and Content	Duration and Frequency, Follow-Up
**Amorim et al., 2019**	**Mobile application** Written, pedometer Telephone call, message	Physical exercise, activity tracker, lessons - Goal setting (behavior) - Problem solving - Action planning - Social support (emotional) - Instruction on how to perform the behavior - Feedback on outcomes of behavior - Graded tasks	6 months 1 face-to-face interview andROMANIA2 calls/monthROMANIAFollow-up: N/A	**Recommendations** Written, brief advice	- Autonomous increase in physical activity - Benefits of physical activity	6 months N/A Follow-up: N/A
**Berman et al., 2009**	**Internet-basedr** Images, audio Email	**Self-care.** Mind-body exercises and lessons - Problem solving - Action planning - Monitoring of behavior by others without feedback - Instruction on how to perform the behavior	6 weeks ≥ 1 session/week Follow-up: N/A	**No intervention** N/A	N/A	N/A N/A Follow-up: N/A
**Boselie et al., 2018**	**Internet-based** Online platform Telephone call, email	**Positive psychology** exercises - Problem solving - Social support (unspecified) - Instruction on how to perform the behavior	8 weeks Call: weeks 1, 3, 5,7 Email: weeks 2, 4, 6, 8 Follow-up: N/A	**Waiting list** N/A	N/A	N/A N/A Follow-up: N/A
**Bossen et al., 2013**	**Internet-based** Written, video Email	Behavior graded activity and exercises - Goal setting (behavior) - Instruction on how to perform the behavior - Graded tasks	9 weeks ≥ 1 session/week Follow-up: 12 weeks	**Waiting list** N/A	N/A	N/A N/A Follow-up: 12 weeks
**Brattberg, 2008**	**Internet-based** Written Telephone call, email	**Self-management.** Emotional Freedom Techniques Self-monitoring of outcome of behavior	8 weeks 1 time/day Follow-up: N/A	**Waiting list**	N/A	N/A N/A Follow-up: N/A
**Bromberg et al., 2012**	**Internet-based +usual care** Written Email	Behavior change, physical activity, lessons - Goal setting (outcome) - Monitoring of behavior by others without feedback - Self-monitoring of behavior - Graded tasks	6 months ≥ 2 sessions/week (first 4 weeks) ≥ 1 sessions/month (final 5 month) Follow-up: N/A	**Usual care** N/A	- Maintain the routine care and self-management effort	N/A N/A Follow-up: N/A
**Buhrman et al., 2004**	**Internet-based** Slideshow, audio Telephone call	**CBT.** Physical and psychological exercises, relaxation - Goal setting (behavior) - Problem solving - Instruction on how to perform the behavior - Self-monitoring of behavior - Graded tasks	6 weeks 1 call/week Follow-up: 3 months	**Waiting list** N/A	N/A	N/A N/A Follow-up: 3 months
**Buhrman et al., 2011**	**Internet-based** Written Email	**CBT.** Physical exercise, relaxation, cognitive skills - Self-monitoring of behavior	8 weeks N/R Follow-up: 12 weeks	**Waiting list** N/A	N/A	N/A N/A Follow-up: 12 weeks
**Calner et al., 2017 and Nordin et al., 2016**	**Internet-based + multimodal rehabilitation** Written, video No contact	Behavior, change, lessons, homework - Goal setting (behavior) - Problem solving - Action planning - Instruction on how to perform the behavior - Reduce negative emotions Physical therapy (i.e., exercises), occupational therapy (i.e., functional training), psychology (i.e., cognitive behavior principles)	6–8 weeks Internet-based: 1 lesson/week Multimodal: 2–3 sessions/week Follow-up: 12 months	**Multimodal rehabilitation** N/A	- Physical therapy (i.e., exercises), occupational therapy (i.e., functional training), psychology (i.e., cognitive behavior principles)	6–8 weeks 2–3 session/week Follow-up: 12 months
**Carpenter et al., 2012**	**Internet-based** Written, images, audio Email	**CBT and pain education.** Lessons, homework, relaxation - Instruction on how to perform the behavior - Reduce negative emotions - Framing/reframing	3 weeks 2 lessons/week Follow-up: 6 weeks	**Waiting list** N/A	N/A	N/A N/A Follow-up: 6 weeks
**Chabbra et al., 2018**	**Mobile application** Written N/R	**Self-management**—Physical exercise - Goal setting (behavior) - Feedback on behavior - Graded tasks	12 weeks N/R Follow-up: N/A	**Usual care** Written	- Pharmacotherapy - Recommendations of physical activity level	12 weeks N/A Follow-up: N/A
**Chiauzzi et al., 2010**	**Internet-based** Written Email	**CBT and self-management.** Lessons, homework - Goal setting (outcome) - Problem solving - Monitoring of behavior by others without feedback - Self-monitoring of behavior	4 weeks 2 sessions/week Follow-up: 6 months	**Recommendations** Written	- Pain information (standard back pain management)	4 weeks N/A Follow-up: 6 months
**Choi et al., 2019**	**Mobile application + NSAIDs** Video, audio N/R	Physical exercise, NSAIDs - Feedback on outcome of behavior	2 months 2–3 times/day Follow-up: 3 months	Physical exercise, NSAIDs Images	Exercise - Feedback on outcome of behavior	2 months 2–3 times/day Follow-up: 3 months
**De Boer et al., 2014**	**Internet-based** Multimedia applications Telephone call, email	**CBT.** Lessons, homework and relaxation - Problem solving - Feedback on behavior - Graded tasks - Distraction Framing/reframing	7 weeks 1 session/week Email: after modules 2, 4, 7, 8 Follow-up: 2 months	**Face-to-face** Book	- CBT. Lessons, homework and relaxation - Problem solving - Graded tasks - Distraction Framing/reframing	7 weeks 1 session/week Follow-up: 2 months
**Dear et al., 2013**	**Internet-based** Written Telephone call	**CBT.** Lessons, homework - Goal setting (behavior) - Graded tasks	8 weeks 1 lesson/7–10 days 1 call/week Follow-up: 3 months	**Waiting list** N/A	N/A	N/A N/A Follow-up: 3 months
**Dear et al., 2015**	**Internet-based** - G1: CBT + Regular online contact - G2: CBT + optimal online contact - G3: CBT Slideshow Telephone call, email	**CBT**. Lessons, homework - Problem solving - Instruction on how to perform the behavior - Behavioral practice - Graded tasks	8 weeks 1 lesson/7–10 days G1: 1 call/week G2: as-needed calls G3: no contact Follow-up: 3 months	**Waiting list** N/A	N/A	N/A N/A Follow-up: 3 months
**Ferwerda et al., 2017**	**Internet-based** Written Email	**CBT.** Lessons, homework - Goal setting (behavior) - Problem solving - Action planning - Instruction on how to perform the behavior - Reduce negative emotions - Distraction - Framing/reframing	17 to 32 weeks 1 email/1–2 weeks Follow-up: 12 months	**Usual care** N/R	- Rheumatological care	N/R N/R Follow-up: 12 months
**Friesen et al., 2017**	**Internet-based** Slideshow Telephone call, email	**CBT.** Lessons, homework - Problem solving - Feedback on perform the behavior - Instruction on how to perform the behavior	8 weeks 1 email and call/week Follow-up: N/A	**Waiting list** N/A	N/A	N/A N/A Follow up: N/A
**Gardner-Nix et al., 2008**	**Videoconferencing** N/R N/R	**Mindfulness** lessons - N/R	10 weeks 2 h/week Follow-up: N/A	G1: **Face-to-face** N/R G2: **Waiting list** N/A	- G1: Mindfulness lessons - G2: N/A	G1: 10 weeks 2 h/week G2: N/A Follow-up: N/A
**Gialanella et al., 2017 and 2020**	**Telephone call** Written, images Telephone call	Physical exercise - Problem solving - Social support (unspecified)	6 months ≥2 calls/month Follow-up: 12 months	**Physical exercise + recommendations** N/R	- Physical exercise - Recommendation to continue exercise at home	6 months N/A Follow-up: 12 months
**Guarino et al., 2018**	**Internet-based + usual care** Written, images, audio Telephone call, email	**CBT.** Lessons, relaxation - Problem solving - Feedback on behavior - Reduce negative emotions - Framing/reframing	12 weeks 2 lessons/week Follow-up: 3 months	**Usual care** N/A	- Pharmacotherapy	12 weeks N/A Follow-up: 3 months
**Heapy et al., 2017**	**Interactive voice response** Written, images, audio, pedometer Telephone call	**CTB.** Lessons, relaxation - Goal setting (outcome) - Feedback on behavior - Graded tasks - Reduce negative emotions	10 weeks 1 call/day Follow-up: 9 months	**Face-to-face** Written, images, audio, pedometer	CBT. Lessons, relaxation - Goal setting (outcome) - Feedback on behavior - Graded tasks - Reduce negative emotions	10 weeks 1 session/week Follow-up: 9 months
**Hedman-Lagerlöf et al., 2018**	**Internet-based** Written Telephone call, message	Lessons, homework, mindfulness - Goal setting (behavior) - Problem solving - Monitoring of behavior by others without feedback - Exposure - Graded tasks	10 weeks 1–3 contacts/week Follow-up: 12 months	**Waiting list** N/A	N/A	N/A N/A Follow-up: 12 months
**Herbert et al., 2017**	**Videoconferencing** Written N/R	**ACT.** Mindfulness, lessons - Goal setting - Information about emotional consequences	8 weeks 1 session/week Follow-up: 6 months	**Face-to-face** Written	ACT. Mindfulness, lessons - Goal setting - Information about emotional consequences	8 weeks 1 session/week Follow-up: 6 months
**Hernando-Garijo et al., 2021**	**Videoconferencing + usual care** Video Video call	Aerobic exercise - Low-impact exercise	15 weeks 2 session/week Follow-up: N/A	**Usual care** N/A	- Maintain pharmacotherapy	15 weeks N/A Follow-up: NA
**Juhlin et al., 2021**	**Internet-based** Digital platform Message	**Person-centered intervention**. Physical and psychological exercises - Goal setting (behavior) - Problem solving - Action planning	6 months 1 contact/week Follow-up: N/A	**Face-to-face** (1 session) N/A	- Person-centered intervention. Physical and psychological exercises	6 months N/A Follow-up: N/A
**Kleiboer et al., 2014**	**Internet-based** Written, audio, video Email	**Behavioral training.** Exercises, lessons, homework, relaxation - Goal setting (behavior) - Problem solving - Instruction on how to perform the behavior	3.6 months on average 8 lessons, 1 lesson/7–10 days Follow-up: N/A	**Waiting list** N/A	N/A	N/A N/A Follow-up: N/A
**Krein et al., 2013**	**Internet-based + pedometer** Written, imagen, digital platform Message, discussion group	**E-community.** Step-count, lessons - Goal setting (outcome) - Feedback on outcome of behavior - Social support (unspecified)	N/R 1 upload data/week Follow-up: 12 month	**Pedometer** N/A	- Step-count - Not receive feedback	N/R 1 upload data/month Follow-up: 12 month
**Lin et al., 2017**	**Internet-based** Written, audio, video Email, message	**ACT.** Lessons, mindfulness - Goal setting (behavior) - Reduce negative emotions	9 weeks 1 session/week Follow-up: 6 months	**Waiting list** N/A	N/A	N/A N/A Follow-up: 6 months
**Lorig et al., 2002**	**Internet-based** Written, video Email discussion group	**E-community.** Physical exercises, lessons - Instruction on how to perform the behavior	6 weeks Frequency determined by user interactions Follow-up: 12 months	**Usual care** N/A	- Maintain usual treatment - Non-health related magazine subscription	6 weeks N/A Follow-up: 12 months
**Lorig et al., 2008**	**Internet-based** Written Email, internet chat	**Self-management.** Physical exercise, lessons, relaxation - Goal setting (behavior) - Problem solving - Action planning - Feedback on behavior - Reduce negative emotions - Distraction	6 weeks ≥3 sessions/week Follow-up: 12 months	**Usual care** N/A	- Maintain usual treatment	6 weeks N/A Follow-up: 12 months
**Maisiak et al., 1996**	**Telephone call** Written Telephone call, email	Counseling strategy - Problem solving - Instruction on how to perform the behavior - Reduce negative emotions	9 months 2 contact/month (first 3 months) 1 contact/month (final 6 months) Follow-up: N/A	**Usual care** N/A	- Maintain usual treatment	9 months N/A Follow-up: N/A
**Moessner et al., 2012**	**Internet-based** N/R Internet guided chat	**Self-monitoring**. Lessons - Self-monitoring of behavior - Behavioral practice/rehearsal	12–15 weeks 1 session/week Follow-up: 6 months	**Usual care** N/A	N/R	12–15 weeks 1 session/week Follow-up: 6 months
**Odole and Ojo, 2013 and 2014**	**Telephone call** N/R Telephone call	Physical therapy: exercises - Self-monitoring of outcome of behavior	6 weeks 3 calls/week Follow-up: N/A	**Face-to-face** N/A	- Physical therapy: exercises	6 weeks 3 sessions/week Follow-up: N/A
**Peters et al., 2017**	**Internet-based** Written Telephone call, email	G1: **Positive psychology.** Psychological exercises - Goal setting (behavior) - Graded tasks - Reduce negative emotions G2: **CBT.** Lessons, homework, relaxation - Problem solving - Action planning - Social support (unspecified) - Framing/reframing	8 weeks 1 lesson/week Call: weeks 1, 3, 5, 7 Email: weeks: 2, 4, 6, 8 Follow-up: 6 months	**Waiting list** N/A	N/A	N/A N/A Follow-up: 6 months
**Petrozzi et al., 2019**	**Internet-based + usual care** Written Telephone call	**CBT.** Lessons, homework - Problem solving - Self-monitoring on behavior - Instruction on how to perform the behavior - Distraction	8 weeks 1 lesson/week 1 call/week Follow-up: 12 months	**Usual care** N/A	- Physical treatment (manual therapy, exercise and/or education) - Recommendation for physical activity	8 weeks 12 sessions (variable frequency) Follow-up: 12 months
**Rickardsson et al., 2021**	**Internet-based** Written, image, audio Telephone call, message	**ACT.** Lessons - Instruction on how to perform the behavior - Feedback on behavior - Graded tasks - Non-specific reward - Distraction	8 weeks 7 sessions/week ≥2 messages/week Follow-up: 12 months	**Waiting list** N/A	- Maintain usual treatment	N/A N/A Follow-up: 12 months
**Ruehlman et al., 2012**	**Internet-based** Written, image Email, message	**Self-management + e-community**. Physical exercise, lessons, homework, relaxation - Goal setting (outcome) - Action planning - Self-monitoring of outcome of behavior - Instruction on how to perform the behavior - Reduce negative emotions	6 weeks N/R Follow-up: 14 weeks	**Usual care** N/A	N/R	6 weeks N/A Follow-up: 14 weeks
**Sander et al., 2020**	**Internet-based + usual care** Written, audio, video Telephone call, email, message	**CBT.** Lessons, homework, relaxation - Problem solving - Action planning - Feedback on behavior - Reduce negative emotions	9 weeks 7 sessions/week Follow-up: 12 months	**Usual care** N/A	- Medical or psychological treatment	9 weeks N/R Follow-up: 12 months
**Schlickler et al., 2020**	**Internet-based + mobile-based** N/R Email, message	**CBT.** Lessons, mindfulness, relaxation - Problem solving - Feedback on behavior - Social support - Non-specific reward - Reduce negative emotions - Framing/reframing	9 weeks 7 lessons/week Follow-up: 6 months	**Waiting list** N/A	N/A	N/A N/A Follow-up: 6 months
**Schulz et al., 2007**	**Internet-based** Multimedia materials Email, forum	Physical exercise, lessons, homework - Problem solving - Instruction on how to perform the behavior	5 months N/R Follow-up: N/A	**No treatment** N/A	N/A	N/A N/A Follow-up: N/A
**Scott et al., 2018**	**Internet-based + usual care** Video Telephone call, email	**ACT.** Lessons - Goal setting (behavior) - Feedback on behavior - Instruction on how to perform the behavior - Monitoring of emotional consequences	5 weeks 2 lesson/week (first 3 weeks), 1 lesson/week (final 2 weeks) Follow-up: 9 months	**Usual care** N/A	- Medical treatment - Instruction on how to perform the behavior	5 weeks N/A Follow-up: 9 months
**Shigaki et al., 2013**	**Internet-based** Slideshow Telephone call, message, online chat	Lessons, homework - Problem solving - Self-monitoring behavior	10 weeks 1 lesson/week 1 call/week Follow-up: N/A	**Waiting list**	N/A	N/A N/A Follow-up: N/A
**Simister al., 2018**	**Internet-based + usual care** Written, audio, video Email	**ACT.** Lessons, homework - Feedback on behavior - Non-specific reward	8 weeks N/R Follow-up: 3 months	**Usual care** N/A	- Maintain usual treatment	8 weeks N/A Follow-up: 3 months
**Smith et al., 2019**	**Internet-based** Written, image, audio, video Telephone call, email	**CBT and self-management.** Multidisciplinary program with physical exercise, lessons, homework, relaxation - Goal setting (behavior and outcome) - Problem solving - Instruction on how to perform the behavior - Graded tasks Multidisciplinary program Physical therapy, psychologist	4 months 2 lessons/month Follow-up: 7 months	**Usual care** N/A	- Maintain usual treatment	4 months N/A Follow-up: 7 months
**Ström et al., 2000**	**Internet-based** Written Email	Lessons, relaxation - Problem solving - Instruction on how to perform the behavior - Feedback on outcome of behavior	6 weeks 1 lesson/week Follow-up: N/A	**Waiting list** N/A	N/A	N/A N/A Follow-up: N/A
**Tavallaei et al., 2018**	**Internet-based** Written N/R	**Mindfulness**-based stress reduction bibliotherapy - Problem solving - Action planning - Distraction	8 weeks 1 lesson/week Follow-up: N/A	**Usual care** N/A	- Pharmacotherapy	8 weeks N/A Follow-up: N/A
**Trompetter et al., 2015**	**Internet-based** Written Email	**ACT.** Lessons, mindfulness - Self-monitoring of behavior - Non-specific reward - Distraction	3 months ≥ 3 h/week Follow-up: 6 months	**Waiting list** N/A	N/A	N/A N/A Follow-up: 6 months
**Trudeau et al., 2015**	**Internet-based** Multimedia materials Telephone call, email	**Self-management**. Lessons - Problem solving - Instruction on how to perform the behavior - Reduce negative emotions	6 months ≥2 sessions/week (1 month) 1 session/month (5 months) Follow-up: N/A	**Waiting list** N/A	N/A	N/A N/A Follow-up: N/A
**Vallejo et al., 2015**	**Internet-based + usual care** Written, images, audio Message	**CBT.** Lessons, homework, relaxation - Problem solving - Feedback on behavior - Reduce negative emotions - Framing/reframing	10 weeks 1 session/week Follow-up: 12 months	G1: **Face-to-face + usual care** Written, images, audio G2: **Usual care** N/A	G1: **CBT.** Lessons, homework, relaxation - Problem solving - Reduce negative emotions - Framing/reframing G2: Pharmacotherapy	10 weeks G1: 1 session/week G2: N/A Follow-up (only G1): 12 months
**Westenberg et al., 2018**	**Internet-based** Written, video N/R	Mindfulness - Reduce negative emotions	60-s video N/R Follow-up: N/A	**Attention control** Written	- Health information	60-s read N/R Follow-up: N/A
**Williams et al., 2010**	**Internet-based + usual care** Written, audio, video No contact	**Self-management.** Lessons, homework, relaxation - Goal setting (behavior) - Problem solving - Self-monitoring of behavior - Social supports (unspecified) - Instruction on how to perform the behavior - Graded tasks - Framing/reframing	6 months N/R Follow-up: N/A	**Usual care**	- Maintain usual treatment from care physician	6 months N/A Follow-up: N/A
**Wilson et al., 2015**	**Internet-based** N/R N/R	**Self-management.** Lessons, exercises, relaxation - Goal setting (outcome) - Self-monitoring or outcome of behavior	8 weeks N/R Follow-up: N/A	**Usual care** N/A	N/A	8 weeks N/R Follow-up: N/A
**Wilson et al., 2018**	**Internet-based** Written Interactive activity	**Self-management.** Lessons, homework - Self-monitoring of behavior - Behavioral practice/rehearsal	8 weeks N/R Follow-up: N/A	**Waiting list** Written	- Educational tips	8 weeks 1 email/week Follow-up: N/A
**Yang et al., 2019**	**Mobile application + face-to-face** N/R Email	**Self-management.** Physical exercise - Self-monitoring of behavior Physiotherapy: manual therapy, electrophsysical therapy, traction	4 weeks Exercises: 4 times/week Physiotherapy: N/R Follow-up: N/A	**Face-to-face** N/A	- Physiotherapy: manual therapy, electrophysical therapy, traction	4 weeks N/R Follow-up: N/A

Abbreviatures: ACT: Acceptance and Commitment therapy; CBT: Cognitive-behavioral therapy; N/A: Not applicable; N/R: Not reported; NSAIDs: Nonsteroidal anti-inflammatory drugs.

## Appendix D. Assessment of the Studies Quality Based on PEDro Scale

**Table:**

Items												
Articles	1	2	3	4	5	6	7	8	9	10	11	Total
Amorim et al., 2019	1	1	1	1	0	0	1	0	1	1	1	**7**
Berman et al., 2009	1	1	0	1	0	0	0	1	0	1	1	**5**
Boselie et al., 2018	0	1	0	1	0	0	0	0	0	1	1	**4**
Bossen et al., 2013	1	1	1	1	0	0	0	0	1	1	1	**6**
Brattberg, 2008	1	1	1	1	0	0	0	1	1	1	1	**7**
Bromberg et al., 2012	1	1	0	1	0	0	0	1	1	1	1	**6**
Buhrman et al., 2004	1	1	0	1	0	0	0	1	0	1	1	**5**
Buhrman et al., 2011	1	1	1	1	0	0	0	1	1	1	1	**7**
Calner et al., 2017	1	1	1	1	0	0	0	0	0	1	1	**5**
Carpenter et al., 2012	1	1	0	1	0	0	0	1	0	1	1	**5**
Chhabra et al., 2018	1	1	1	1	0	0	1	1	1	1	1	**8**
Chiauzzi et al., 2010	1	1	0	1	0	0	0	1	1	1	1	**6**
Choi et al., 2019	1	1	1	1	0	0	0	1	1	1	1	**7**
De Boer et al., 2014	1	1	0	1	0	0	0	1	0	1	1	**5**
Dear et al., 2013	1	1	0	1	0	0	0	1	0	1	1	**5**
Dear et al., 2015	1	1	1	1	0	0	0	1	0	1	1	**6**
Ferwerda et al., 2017	1	1	1	1	0	0	0	1	1	1	1	**7**
Friesen et al., 2017	1	1	1	1	0	0	0	1	0	1	1	**6**
Gardner-Nix et al., 2008	1	1	0	1	0	0	0	1	0	1	1	**5**
Gialanella et al., 2017	1	1	0	1	0	0	0	1	0	1	1	**5**
Gialanella et al., 2020	1	1	1	1	0	0	0	1	0	1	1	**6**
Guarino et al., 2018	1	1	0	1	0	0	0	1	0	1	1	**5**
Heapy et al., 2017	1	1	1	1	0	0	0	0	1	1	1	**6**
Hedman-Lagerlöf et al., 2018	1	1	1	1	0	0	0	1	0	1	1	**6**
Herbert et al., 2017	1	1	0	1	0	0	1	1	1	1	1	**7**
Hernando-Garijo et al., 2021	1	1	0	1	0	0	1	1	1	1	1	**7**
Juhlin et al., 2021	1	1	1	1	0	0	0	0	1	1	1	**6**
Kleiboer et al., 2014	1	1	1	1	0	0	0	1	1	1	1	**7**
Krein et al., 2013	1	1	1	1	0	0	0	1	1	1	1	**7**
Lin et al., 2017	1	1	1	1	0	0	0	0	1	1	1	**6**
Lorig et al., 2002	1	1	0	1	0	0	0	1	1	1	1	**6**
Lorig et al., 2008	1	1	0	1	0	0	0	0	1	1	1	**5**
Maisiak et al., 1996	1	1	0	1	0	0	1	1	0	1	1	**6**
Moessner et al., 2012	1	1	0	1	0	0	0	0	1	1	1	**5**
Nordin et al., 2016	1	1	1	1	0	0	0	1	1	1	1	**7**
Odole and Ojo, 2013	1	1	0	1	0	0	0	1	0	1	1	**5**
Odole and Ojo, 2014	1	1	0	1	0	0	0	1	0	1	1	**5**
Peters et al., 2017	1	1	0	1	0	0	0	0	1	1	1	**5**
Petrozzi et al., 2019	1	1	1	1	0	0	0	1	1	1	1	**7**
Rickardsson et al., 2021	1	1	1	1	0	0	0	1	1	1	1	**7**
Ruehlman et al., 2012	1	1	0	1	0	0	0	0	1	1	1	**5**
Sander et al., 2020	1	1	1	1	0	0	1	0	1	1	1	**7**
Schlicker et al., 2020	1	1	0	1	0	0	0	1	1	1	1	**6**
Schulz et al., 2007	1	1	0	1	0	0	0	0	1	1	1	**5**
Scott et al., 2018	1	1	1	1	0	0	0	1	1	1	1	**7**
Shigaki et al., 2013	1	1	0	0	0	0	0	1	0	1	1	**4**
Simister et al., 2018	1	1	1	1	0	0	0	1	1	1	1	**7**
Smith et al., 2019	1	1	0	1	0	0	1	0	1	1	1	**6**
Ström et al., 2000	1	1	0	1	0	0	0	0	1	1	1	**5**
Tavallaei et al., 2018	1	1	0	0	0	0	0	1	0	1	1	**4**
Trompetter et al., 2015	1	1	0	1	0	0	0	1	1	1	1	**6**
Trudeau et al., 2015	1	1	1	1	0	0	0	1	1	1	1	**7**
Vallejo et al., 2015	1	1	0	1	0	0	0	1	1	1	1	**6**
Westenberg et al., 2018	1	1	0	1	1	0	0	1	1	1	1	**7**
Williams et al., 2010	1	1	1	1	0	0	0	1	1	1	1	**7**
Wilson et al., 2015	1	1	0	1	0	0	0	0	1	1	1	**5**
Wilson et al., 2018	1	1	0	1	0	0	0	0	0	1	1	**4**
Yang et al., 2019	1	1	1	1	0	0	0	0	1	1	1	**6**

Notes: 1: subject choice criteria are specified; 2: random assignment of subjects to groups; 3: hidden assignment; 4: groups were similar at baseline; 5: all subjects were blinded; 6: all therapists were blinded; 7: all evaluators were blinded; 8: measures of at least one of the key outcomes were obtained from more than 85% of baseline subjects; 9: intention-to-treat analysis was performed; 10: results from statistical comparisons between groups were reported for at least one key outcome; 11: the study provides point and variability measures for at least one key outcome. 1: item 1 does not contribute to the final score.

## Appendix F. Statistical Exploration of Heterogeneity, Outliers, Robustness and Publication Bias for the Pain Intensity Variable

Forest plot with all the studies

Influence analyses of all the studies

Leave-one-out figure of all the studies

Contour-enhanced funnel plot of the studies included in the sensitivity analysis

Doi plot and LFK index of the studies included in the sensitivity analysis

Drapery plot of the studies included in the sensitivity analysis

Contour-enhanced funnel plot of the studies included in the sensitivity analysis and the studies filled to adjust for publication bias

Forest plot of the studies included in the sensitivity analysis and the studies filled to adjust for publication bias

## Appendix G. Statistical Exploration of Heterogeneity, Outliers, Robustness and Publication Bias for the Pain Intensity Variable (e-BMT vs. In-Person BMT)

Influence Analyses of all the studies

Leave-one-out figure of all the studies

Contour-enhanced funnel plot of all the studies

Doi plot and LFK index of all the studies

Drapery plot of all the studies

Contour-enhanced funnel plot of the studies included in the sensitivity analysis and the studies filled to adjust for publication bias. The Trim and Fill Method did not add any study.

Forest plot of the studies included in the sensitivity analysis and the studies filled to adjust for publication bias. The Trim and Fill Method did not add any study.

## Appendix H. Statistical Exploration of Heterogeneity, Outliers, Robustness and Publication Bias for the Pain Interference Variable

Influence Analyses of all the studies

Leave-one-out figure of all the studies

Contour-enhanced funnel plot of all the studies

Doi plot and LFK index of all the studies

Drapery plot of all the studies

## Appendix I. Statistical Exploration of Heterogeneity, Outliers, Robustness and Publication Bias for the Kinesiophobia Variable

Forest plot of all studies

Influence analyses of all studies

Leave-one-out figure of all studies

Contour-enhanced funnel plot of the studies included in the sensitivity analysis

Doi plot and LFK index of the studies included in the sensitivity analysis

Drapery plot of all studies of the studies included in the sensitivity analysis

Contour-enhanced funnel plot of the studies included in the sensitivity analysis and the studies filled to adjust for publication bias

Forest plot of the studies included in the sensitivity analysis and the studies filled to adjust for publication bias

## Appendix J. Statistical Exploration of Heterogeneity, Outliers, Robustness and Publication Bias for the Catastrophizing Variable

Forest plot of all studies

Influence analyses of all the studies for the Overall score subgroup

Leave-one-out figure of all the studies for the Overall score subgroup

Influence Analyses of all the studies for the Helplessness score subgroup

Leave-one-out figure of all the studies for the Helplessness score subgroup

Influence analyses of all the studies for the Magnification score subgroup

Leave-one-out figure of all the studies for the Magnification score subgroup

Influence Analyses of all the studies for the Rumination score subgroup

Leave-one-out figure of all the studies for the Rumination score subgroup

Contour-enhanced funnel plot of the studies included in the sensitivity analysis

Doi plot and LFK index of the studies included in the sensitivity analysis

Drapery plot of the studies included in the sensitivity analysis

## Appendix K. Statistical Exploration of Heterogeneity, Outliers, Robustness and Publication Bias for the Self-Efficacy

Forest plot of all studies

Influence analyses of all studies

Leave-one-out figure of all studies

Contour-enhanced funnel plot of the sensitivity analysis

Doi plot and LFK index of all studies

Drapery plot of all studies

Contour-enhanced funnel plot of the studies included in the sensitivity analysis and the studies filled to adjust for publication bias

Forest plot of the studies included in the sensitivity analysis and the studies filled to adjust for publication bias

## References

[1] Wade D.T. (2020). Rehabilitation after COVID-19: An evidence-based approach. Clin. Med..

[2] Mechanic O.J., Persaud Y., Kimball A.B. (2021). Telehealth Systems. Treasure Island.

[3] Mead N., Bower P. (2000). Patient-centredness: A conceptual framework and review of the empirical literature. Soc. Sci. Med..

[4] Pear J.J., Martin G.L., Seel N.M. (2012). Behavior Modification, Behavior Therapy, Applied Behavior Analysis and Learning. Encyclopedia of the Sciences of Learning.

[5] Moyer R., Ikert K., Long K., Marsh J. (2017). The Value of Preoperative Exercise and Education for Patients Undergoing Total Hip and Knee Arthroplasty: A Systematic Review and Meta-Analysis. JBJS Rev..

[6] Bunzli S., Gillham D., Esterman A. (2011). Physiotherapy-provided operant conditioning in the management of low back pain disability: A systematic review. Physiother. Res. Int..

[7] Willett M., Duda J., Fenton S., Gautrey C., Greig C., Rushton A. (2019). Effectiveness of behaviour change techniques in physiotherapy interventions to promote physical activity adherence in lower limb osteoarthritis patients: A systematic review. PLoS ONE.

[8] Meade L.B., Bearne L.M., Sweeney L.H., Alageel S.H., Godfrey E.L. (2019). Behaviour change techniques associated with adherence to prescribed exercise in patients with persistent musculoskeletal pain: Systematic review. Br. J. Health Psychol..

[9] Marley J., Tully M.A., Porter-Armstrong A., Bunting B., O’Hanlon J., Atkins L., Howes S., McDonough S.M. (2017). The effectiveness of interventions aimed at increasing physical activity in adults with persistent musculoskeletal pain: A systematic review and meta-analysis. BMC Musculoskelet. Disord..

[10] Button K., Roos P.E., Spasić I., Adamson P., van Deursen R.W.M. (2015). The clinical effectiveness of self-care interventions with an exercise component to manage knee conditions: A systematic review. Knee.

[11] Leite H., Hodgkinson I.R., Gruber T. (2020). New development: ‘Healing at a distance’—Telemedicine and COVID-19. Public Money Manag..

[12] Smith A.C., Thomas E., Snoswell C.L., Haydon H., Mehrotra A., Clemensen J., Caffery L.J. (2020). Telehealth for global emergencies: Implications for coronavirus disease 2019 (COVID-19). J. Telemed. Telecare.

[13] Lacasse A., Pagé M.G., Dassieu L., Sourial N., Janelle-Montcalm A., Dorais M., Nguena Nguefack H.L., Godbout-Parent M., Hudspith M., Moor G. (2021). Impact of the COVID-19 pandemic on the pharmacological, physical, and psychological treatments of pain: Findings from the Chronic Pain & COVID-19 Pan-Canadian Study. Pain Rep..

[14] Eccleston C., Blyth F.M., Dear B.F., Fisher E.A., Keefe F.J., Lynch M.E., Palermo T.M., Reid M.C., de C Williams A.C. (2020). Managing patients with chronic pain during the COVID-19 outbreak: Considerations for the rapid introduction of remotely supported (eHealth) pain management services. Pain.

[15] Nieto R., Pardo R., Sora B., Feliu-Soler A., Luciano J.V. (2020). Impact of COVID-19 Lockdown Measures on Spanish People with Chronic Pain: An Online Study Survey. J. Clin. Med..

[16] Karos K., McParland J.L., Bunzli S., Devan H., Hirsh A., Kapos F.P., Keogh E., Moore D., Tracy L.M., Ashton-James C.E. (2020). The social threats of COVID-19 for people with chronic pain. Pain.

[17] Varangot-Reille C., Suso-Martí L., Romero-Palau M., Suárez-Pastor P., Cuenca-Martínez F. (2021). Effects of Different Therapeutic Exercise Modalities on Migraine or Tension-Type Headache: A Systematic Review and Meta-Analysis with a Replicability Analysis. J. Pain.

[18] Coulter I.D., Crawford C., Hurwitz E.L., Vernon H., Khorsan R., Suttorp Booth M., Herman P.M. (2018). Manipulation and mobilization for treating chronic low back pain: A systematic review and meta-analysis. Spine J..

[19] Panunzio A., Tafuri A., Mazzucato G., Cerrato C., Orlando R., Pagliarulo V., Antonelli A., Cerruto M.A. (2022). Botulinum Toxin-A Injection in Chronic Pelvic Pain Syndrome Treatment: A Systematic Review and Pooled Meta-Analysis. Toxins.

[20] Varangot-Reille C., Suso-Martí L., Dubuis V., Cuenca-Martínez F., Blanco-Díaz M., Salar-Andreu C., Casaña J., Calatayud J. (2022). Exercise and Manual Therapy for the Treatment of Primary Headache: An Umbrella and Mapping Review. Phys. Ther..

[21] Dupeyron A., Ribinik P., Gélis A., Genty M., Claus D., Hérisson C., Coudeyre E. (2011). Education in the management of low back pain: Literature review and recall of key recommendations for practice. Ann. Phys. Rehabil. Med..

[22] Wood L., Hendrick P.A. (2019). A systematic review and meta-analysis of pain neuroscience education for chronic low back pain: Short-and long-term outcomes of pain and disability. Eur. J. Pain.

[23] Hall M., Dobson F., Plinsinga M., Mailloux C., Starkey S., Smits E., Hodges P., Vicenzino B., Schabrun S.M., Masse-Alarie H. (2020). Effect of exercise on pain processing and motor output in people with knee osteoarthritis: A systematic review and meta-analysis. Osteoarthr. Cartil..

[24] Du S., Hu L., Dong J., Xu G., Chen X., Jin S., Zhang H., Yin H. (2017). Self-management program for chronic low back pain: A systematic review and meta-analysis. Patient Educ. Couns..

[25] Ariza-Mateos M.J., Cabrera-Martos I., Prados-Román E., Granados-Santiago M., Rodríguez-Torres J., Carmen Valenza M. (2020). A systematic review of internet-based interventions for women with chronic pain. Br. J. Occup. Ther..

[26] White V., Linardon J., Stone J.E., Holmes-Truscott E., Olive L., Mikocka-Walus A., Hendrieckx C., Evans S., Speight J. (2022). Online psychological interventions to reduce symptoms of depression, anxiety, and general distress in those with chronic health conditions: A systematic review and meta-analysis of randomized controlled trials. Psychol. Med..

[27] Dario A.B., Moreti Cabral A., Almeida L., Ferreira M.L., Refshauge K., Simic M., Pappas E., Ferreira P.H. (2017). Effectiveness of telehealth-based interventions in the management of non-specific low back pain: A systematic review with meta-analysis. Spine J..

[28] Page M.J., McKenzie J.E., Bossuyt P.M., Boutron I., Hoffmann T.C., Mulrow C.D., Shamseer L., Tetzlaff J.M., Akl E.A., Brennan S.E. (2021). The PRISMA 2020 statement: An updated guideline for reporting systematic reviews. BMJ.

[29] Stone P.W. (2002). Popping the (PICO) question in research and evidence-based practice. Appl. Nurs. Res..

[30] Shariff S.Z., Bejaimal S.A., Sontrop J.M., Iansavichus A.V., Haynes R.B., Weir M.A., Garg A.X. (2013). Retrieving clinical evidence: A comparison of PubMed and Google Scholar for quick clinical searches. J. Med. Internet Res..

[31] Haddaway N.R., Collins A.M., Coughlin D., Kirk S. (2015). The Role of Google Scholar in Evidence Reviews and Its Applicability to Grey Literature Searching. PLoS ONE.

[32] Moher D., Pham B., Jones A., Cook D.J., Jadad A.R., Moher M., Tugwell P., Klassen T.P. (1998). Does quality of reports of randomised trials affect estimates of intervention efficacy reported in meta-analyses?. Lancet.

[33] Ouzzani M., Hammady H., Fedorowicz Z., Elmagarmid A. (2016). Rayyan—A web and mobile app for systematic reviews. Syst. Rev..

[34] Furlan A.D., Pennick V., Bombardier C., van Tulder M. (2009). 2009 updated method guidelines for systematic reviews in the Cochrane Back Review Group. Spine.

[35] Higgins J., Green S. (2008). Cochrane Handbook for Systematic Reviews of Interventions.

[36] Sterne J.A.C., Savović J., Page M.J., Elbers R.G., Blencowe N.S., Boutron I., Cates C.J., Cheng H.-Y., Corbett M.S., Eldridge S.M. (2019). RoB 2: A revised tool for assessing risk of bias in randomised trials. BMJ.

[37] De Morton N.A. (2009). The PEDro scale is a valid measure of the methodological quality of clinical trials: A demographic study. Aust. J. Physiother..

[38] Hariohm K., Prakash V., Saravankumar J. (2015). Quantity and quality of randomized controlled trials published by Indian physiotherapists. Perspect. Clin. Res..

[39] Landis J.R., Koch G.G. (1977). An Application of Hierarchical Kappa-type Statistics in the Assessment of Majority Agreement among Multiple Observers. Biometrics.

[40] Guyatt G.H., Oxman A.D., Vist G.E., Kunz R., Falck-Ytter Y., Alonso-Coello P., Schünemann H.J. (2008). GRADE Working Group GRADE: An emerging consensus on rating quality of evidence and strength of recommendations. BMJ.

[41] Andrews J., Guyatt G., Oxman A.D., Alderson P., Dahm P., Falck-Ytter Y., Nasser M., Meerpohl J., Post P.N., Kunz R. (2013). GRADE guidelines: 14. Going from evidence to recommendations: The significance and presentation of recommendations. J. Clin. Epidemiol..

[42] Balshem H., Helfand M., Schünemann H.J., Oxman A.D., Kunz R., Brozek J., Vist G.E., Falck-Ytter Y., Meerpohl J., Norris S. (2011). GRADE guidelines: 3. Rating the quality of evidence. J. Clin. Epidemiol..

[43] Sanabria A.J., Rigau D., Rotaeche R., Selva A., Marzo-Castillejo M., Alonso-Coello P. (2015). GRADE: Methodology for formulating and grading recommendations in clinical practice. Aten. Primaria.

[44] Harrer M., Cuijpers P., Furukawa T.A., Ebert D.D. (2021). Doing Meta-Analysis with R: A Hands-On Guide.

[45] Hedges L. (1982). Estimation of effect size from a series of independent experiments. Psychol. Bull..

[46] Hopkins W.G., Marshall S.W., Batterham A.M., Hanin J. (2009). Progressive statistics for studies in sports medicine and exercise science. Med. Sci. Sports Exerc..

[47] Higgins J.P., Li T., Deeks J.J. 6.5.2.3 Obtaining Standard Deviations from Standard Errors, Confidence Intervals, t Statistics and *p* Values for Differences in Means. https://handbook-5-1.cochrane.org/chapter_7/7_7_3_3_obtaining_standard_deviations_from_standard_errors.htm.

[48] Viechtbauer W. (2005). Bias and Efficiency of Meta-Analytic Variance Estimators in the Random-Effects Model. J. Educ. Behav. Stat..

[49] Veroniki A.A., Jackson D., Viechtbauer W., Bender R., Bowden J., Knapp G., Kuss O., Higgins J.P.T., Langan D., Salanti G. (2016). Methods to estimate the between-study variance and its uncertainty in meta-analysis. Res. Synth. Methods.

[50] Knapp G., Hartung J. (2003). Improved tests for a random effects meta-regression with a single covariate. Stat. Med..

[51] Sidik K., Jonkman J.N. (2002). A simple confidence interval for meta-analysis. Stat. Med..

[52] Sullivan M.J.L., Bishop S.R., Pivik J. (1995). The Pain Catastrophizing Scale: Development and validation. Psychol. Assess..

[53] Borenstein M., Higgins J.P.T. (2013). Meta-Analysis and Subgroups. Prev. Sci..

[54] Hoaglin D. (2016). Misunderstandings about Q and “Cochran’s Q test” in meta-analysis. Stat. Med..

[55] Borenstein M., Higgins J.P.T., Hedges L.V., Rothstein H.R. (2017). Basics of meta-analysis: I(2) is not an absolute measure of heterogeneity. Res. Synth. Methods.

[56] IntHout J., Ioannidis J.P.A., Rovers M.M., Goeman J.J. (2016). Plea for routinely presenting prediction intervals in meta-analysis. BMJ Open.

[57] Viechtbauer W., Cheung M.W.-L. (2010). Outlier and influence diagnostics for meta-analysis. Res. Synth. Methods.

[58] Rücker G., Schwarzer G. (2021). Beyond the forest plot: The drapery plot. Res. Synth. Methods.

[59] Doi S.A. (2018). Rendering the Doi plot properly in meta-analysis. Int. J. Evid.-Based. Healthc..

[60] Furuya-Kanamori L., Barendregt J.J., Doi S.A.R. (2018). A new improved graphical and quantitative method for detecting bias in meta-analysis. Int. J. Evid. Based. Healthc..

[61] Duval S., Tweedie R. (2000). Trim and fill: A simple funnel-plot-based method of testing and adjusting for publication bias in meta-analysis. Biometrics.

[62] Amorim A.B., Pappas E., Simic M., Ferreira M.L., Jennings M., Tiedemann A., Carvalho-E-Silva A.P., Caputo E., Kongsted A., Ferreira P.H. (2019). Integrating Mobile-health, health coaching, and physical activity to reduce the burden of chronic low back pain trial (IMPACT): A pilot randomised controlled trial. BMC Musculoskelet. Disord..

[63] Berman R.L.H., Iris M.A., Bode R., Drengenberg C. (2009). The Effectiveness of an Online Mind-Body Intervention for Older Adults with Chronic Pain. J. Pain.

[64] Chiauzzi E., Pujol L.A., Wood M., Bond K., Black R., Yiu E., Zacharoff K. (2010). PainACTION-Back Pain: A self-management website for people with chronic back pain. Pain Med..

[65] Dear B.F., Titov N., Perry K.N., Johnston L., Wootton B.M., Terides M.D., Rapee R.M., Hudson J.L. (2013). The Pain Course: A randomised controlled trial of a clinician-guided Internet-delivered cognitive behaviour therapy program for managing chronic pain and emotional well-being. Pain.

[66] Dear B.F., Gandy M., Karin E., Staples L.G., Johnston L., Fogliati V.J., Wootton B.M., Terides M.D., Kayrouz R., Perry K.N. (2015). The Pain Course: A randomised controlled trial examining an internet-delivered pain management program when provided with different levels of clinician support. Pain.

[67] De Boer M.J., Versteegen G.J., Vermeulen K.M., Sanderman R., Struys M.M.R.F. (2014). A randomized controlled trial of an Internet-based cognitive-behavioural intervention for non-specific chronic pain: An effectiveness and cost-effectiveness study. Eur. J. Pain.

[68] Ferwerda M., Van Beugen S., Van Middendorp H., Spillekom-Van Koulil S., Donders A.R.T., Visser H., Taal E., Creemers M.C.W., Van Riel P.C.L.M., Evers A.W.M. (2017). A tailored-guided internet-based cognitive-behavioral intervention for patients with rheumatoid arthritis as an adjunct to standard rheumatological care: Results of a randomized controlled trial. Pain.

[69] Friesen L.N., Hadjistavropoulos H.D., Schneider L.H., Alberts N.M., Titov N., Dear B.F. (2017). Examination of an internet-delivered cognitive behavioural pain management course for adults with fibromyalgia: A randomized controlled trial. Pain.

[70] Gardner-Nix J., Backman S., Barbati J., Grummitt J. (2008). Evaluating distance education of a mindfulness-based meditation programme for chronic pain management. J. Telemed. Telecare.

[71] Gialanella B., Ettori T., Faustini S., Baratti D., Bernocchi P., Comini L., Scalvini S. (2017). Home-Based Telemedicine in Patients with Chronic Neck Pain. Am. J. Phys. Med. Rehabil..

[72] Gialanella B., Comini L., Olivares A., Gelmini E., Ubertini E., Grioni G. (2020). Pain, disability and adherence to home exercises in patients with chronic neck pain: Long term effects of phone surveillance. A randomized controlled study. Eur. J. Phys. Rehabil. Med..

[73] Guarino H., Fong C., Marsch L.A., Acosta M.C., Syckes C., Moore S.K., Cruciani R.A., Portenoy R.K., Turk D.C., Rosenblum A. (2018). Web-based cognitive behavior therapy for chronic pain patients with aberrant drug-related behavior: Outcomes from a randomized controlled trial. Pain Med..

[74] Bossen D., Veenhof C., van Beek K.E., Spreeuwenberg P.M., Dekker J., de Bakker D.H. (2013). Effectiveness of a web-based physical activity intervention in patients with knee and/or hip osteoarthritis: Randomized controlled trial. J. Med. Internet Res..

[75] Heapy A.A., Higgins D.M., Goulet J.L., La Chappelle K.M., Driscoll M.A., Czlapinski R.A., Buta E., Piette J.D., Krein S.L., Kerns R.D. (2017). Interactive voice response-based self-management for chronic back Pain: The Copes noninferiority randomized trial. JAMA Intern. Med..

[76] Herbert M.S., Afari N., Liu L., Heppner P., Rutledge T., Williams K., Eraly S., VanBuskirk K., Nguyen C., Bondi M. (2017). Telehealth Versus In-Person Acceptance and Commitment Therapy for Chronic Pain: A Randomized Noninferiority Trial. J. Pain.

[77] Hernando-Garijo I., Ceballos-Laita L., Mingo-Gómez M.T., Medrano-De-la-fuente R., Estébanez-De-miguel E., Martínez-Pérez M.N., Jiménez-Del-barrio S. (2021). Immediate effects of a telerehabilitation program based on aerobic exercise in women with fibromyalgia. Int. J. Environ. Res. Public Health.

[78] Juhlin S., Bergenheim A., Gjertsson I., Larsson A., Mannerkorpi K. (2021). Physical activity with person-centred guidance supported by a digital platform for persons with chronic widespread pain: A randomized controlled trial. J. Rehabil. Med..

[79] Kleiboer A., Sorbi M., van Silfhout M., Kooistra L., Passchier J. (2014). Short-term effectiveness of an online behavioral training in migraine self-management: A randomized controlled trial. Behav. Res. Ther..

[80] Krein S.L., Kadri R., Hughes M., Kerr E.A., Piette J.D., Holleman R., Kim H.M., Richardson C.R. (2013). Pedometer-based internet-mediated intervention for adults with chronic low back pain: Randomized controlled trial. J. Med. Internet Res..

[81] Lin J., Paganini S., Sander L., Lüking M., Daniel Ebert D., Buhrman M., Andersson G., Baumeister H. (2017). An Internet-based intervention for chronic pain—A three-arm randomized controlled study of the effectiveness of guided and unguided acceptance and commitment therapy. Dtsch. Arztebl. Int..

[82] Lorig K.R., Laurent D.D., Deyo R.A., Marnell M.E., Minor M.A., Ritter P.L. (2002). Can a back pain e-mail discussion group improve health status and lower health care costs? A randomized study. Arch. Intern. Med..

[83] Lorig K.R., Ritter P.L., Laurent D.D., Plant K. (2008). The internet-based arthritis self-management program: A one-year randomized trial for patients with arthritis or fibromyalgia. Arthritis Care Res..

[84] Maisiak R., Austin J., Heck L. (1996). Health outcomes of two telephone interventions for patients with rheumatoid arthritis or osteoarthritis. Arthritis Rheum..

[85] Buhrman M., Fältenhag S., Ström L., Andersson G. (2004). Controlled trial of Internet-based treatment with telephone support for chronic back pain. Pain.

[86] Moessner M., Schiltenwolf M., Neubauer E. (2012). Internet-based aftercare for patients with back pain—A pilot study. Telemed. e-Health.

[87] Choi Y., Nam J., Yang D., Jung W., Lee H.R., Kim S.H. (2019). Effect of smartphone application-supported self-rehabilitation for frozen shoulder: A prospective randomized control study. Clin. Rehabil..

[88] Odole A.C., Ojo O.D. (2013). A Telephone-based Physiotherapy Intervention for Patients with Osteoarthritis of the Knee. Int. J. Telerehabilit..

[89] Odole A.C., Ojo O.D. (2014). Is telephysiotherapy an option for improved quality of life in patients with osteoarthritis of the knee?. Int. J. Telemed. Appl..

[90] Peters M.L., Smeets E., Feijge M., Van Breukelen G., Andersson G., Buhrman M., Linton S.J. (2017). Happy Despite Pain: A Randomized Controlled Trial of an 8-Week Internet-delivered Positive Psychology Intervention for Enhancing Well-being in Patients with Chronic Pain. Clin. J. Pain.

[91] Petrozzi M.J., Leaver A., Ferreira P.H., Rubinstein S.M., Jones M.K., Mackey M.G. (2019). Addition of MoodGYM to physical treatments for chronic low back pain: A randomized controlled trial. Chiropr. Man. Ther..

[92] Rickardsson J., Gentili C., Holmström L., Zetterqvist V., Andersson E., Persson J., Lekander M., Ljótsson B., Wicksell R.K. (2021). Internet-delivered acceptance and commitment therapy as microlearning for chronic pain: A randomized controlled trial with 1-year follow-up. Eur. J. Pain.

[93] Ruehlman L.S., Karoly P., Enders C. (2012). A randomized controlled evaluation of an online chronic pain self management program. Pain.

[94] Sander L.B., Paganini S., Terhorst Y., Schlicker S., Lin J., Spanhel K., Buntrock C., Ebert D.D., Baumeister H. (2020). Effectiveness of a Guided Web-Based Self-help Intervention to Prevent Depression in Patients with Persistent Back Pain: The PROD-BP Randomized Clinical Trial. JAMA Psychiatry.

[95] Schlicker S., Baumeister H., Buntrock C., Sander L., Paganini S., Lin J., Berking M., Lehr D., Ebert D.D. (2020). A Web- and mobile-Based intervention for comorbid, recurrent depression in patients with chronic back pain on sick leave (get.back): Pilot randomized controlled trial on feasibility, user satisfaction, and effectiveness. JMIR Ment. Health.

[96] Buhrman M., Nilsson-Ihrfelt E., Jannert M., Ström L., Andersson G. (2011). Guided internet-based cognitive behavioural treatment for chronic back pain reduces pain catastrophizing: A randomized controlled trial. J. Rehabil. Med..

[97] Schulz P.J., Rubinell S., Hartung U. (2007). An internet-based approach to enhance self-management of chronic low back pain in the Italian-speaking population of Switzerland: Results from a pilot study. Int. J. Public Health.

[98] Simister H.D., Tkachuk G.A., Shay B.L., Vincent N., Pear J.J., Skrabek R.Q. (2018). Randomized Controlled Trial of Online Acceptance and Commitment Therapy for Fibromyalgia. J. Pain.

[99] Smith J., Faux S.G., Gardner T., Hobbs M.J., James M.A., Joubert A.E., Kladnitski N., Newby J.M., Schultz R., Shiner C.T. (2019). Reboot Online: A Randomized Controlled Trial Comparing an Online Multidisciplinary Pain Management Program with Usual Care for Chronic Pain. Pain Med..

[100] Ström L., Pettersson R., Andersson G. (2000). A controlled trial of self-help treatment of recurrent headache conducted via the Internet. J. Consult. Clin. Psychol..

[101] Tavallaei V., Rezapour-Mirsaleh Y., Rezaiemaram P., Saadat S.H. (2018). Mindfulness for female outpatients with chronic primary headaches: An internet-based bibliotherapy. Eur. J. Transl. Myol..

[102] Trompetter H.R., Bohlmeijer E.T., Veehof M.M., Schreurs K.M.G. (2015). Internet-based guided self-help intervention for chronic pain based on Acceptance and Commitment Therapy: A randomized controlled trial. J. Behav. Med..

[103] Trudeau K.J., Pujol L.A., DasMahapatra P., Wall R., Black R.A., Zacharoff K. (2015). A randomized controlled trial of an online self-management program for adults with arthritis pain. J. Behav. Med..

[104] Vallejo M.A., Ortega J., Rivera J., Comeche M.I., Vallejo-Slocker L. (2015). Internet versus face-to-face group cognitive-behavioral therapy for fibromyalgia: A randomized control trial. J. Psychiatr. Res..

[105] Westenberg R.F., Zale E.L., Heinhuis T.J., Ozkan S., Nazzal A., Lee S.G., Chen N.C., Vranceanu A.M. (2018). Does a brief mindfulness exercise improve outcomes in upper extremity patients? A randomized controlled trial. Clin. Orthop. Relat. Res..

[106] Wilson M., Roll J.M., Corbett C., Barbosa-Leiker C. (2015). Empowering Patients with Persistent Pain Using an Internet-based Self-Management Program. Pain Manag. Nurs..

[107] Bromberg J., Wood M.E., Black R.A., Surette D.A., Zacharoff K.L., Chiauzzi E.J. (2012). A randomized trial of a web-based intervention to improve migraine self-management and coping. Headache.

[108] Wilson M., Finlay M., Orr M., Barbosa-Leiker C., Sherazi N., Roberts M.L.A., Layton M., Roll J.M. (2018). Engagement in online pain self-management improves pain in adults on medication-assisted behavioral treatment for opioid use disorders. Addict. Behav..

[109] Yang J., Wei Q., Ge Y., Meng L., Zhao M. (2019). Smartphone-Based Remote Self-Management of Chronic Low Back Pain: A Preliminary Study. J. Healthc. Eng..

[110] Hedman-Lagerlöf M., Hedman-Lagerlöf E., Axelsson E., Ljotsson B., Engelbrektsson J., Hultkrantz S., Lundbäck K., Björkander D., Wicksell R.K., Flink I. (2018). Internet-Delivered Exposure Therapy for Fibromyalgia a Randomized Controlled Trial. Clin. J. Pain.

[111] Williams D.A., Kuper D., Segar M., Mohan N., Sheth M., Clauw D.J. (2010). Internet-enhanced management of fibromyalgia: A randomized controlled trial. Pain.

[112] Shigaki C.L., Smarr K.L., Siva C., Ge B., Musser D., Johnson R. (2013). RAHelp: An online intervention for individuals with rheumatoid arthritis. Arthritis Care Res..

[113] Brattberg G. (2008). Self-administered EFT (Emotional Freedom Techniques) in individuals with fibromyalgia: A randomized trial. Integr. Med..

[114] Calner T., Nordin C., Eriksson M.K., Nyberg L., Gard G., Michaelson P. (2017). Effects of a self-guided, web-based activity programme for patients with persistent musculoskeletal pain in primary healthcare: A randomized controlled trial. Eur. J. Pain.

[115] Nordin C.A., Michaelson P., Gard G., Eriksson M.K. (2016). Effects of the web behavior change program for activity and multimodal pain rehabilitation: Randomized controlled trial. J. Med. Internet Res..

[116] Carpenter K.M., Stoner S.A., Mundt J.M., Stoelbc B. (2012). An online self-help CBT intervention for chronic lower back pain. Clin. J. Pain.

[117] Chhabra H.S., Sharma S., Verma S. (2018). Smartphone app in self-management of chronic low back pain: A randomized controlled trial. Eur. Spine J..

[118] Boselie J.J.L.M., Vancleef L.M.G., Peters M.L. (2018). Filling the glass: Effects of a positive psychology intervention on executive task performance in chronic pain patients. Eur. J. Pain.

[119] Scott W., Chilcot J., Guildford B., Daly-Eichenhardt A., McCracken L.M. (2018). Feasibility randomized-controlled trial of online Acceptance and Commitment Therapy for patients with complex chronic pain in the United Kingdom. Eur. J. Pain.

[120] Michie S., Richardson M., Johnston M., Abraham C., Francis J., Hardeman W., Eccles M.P., Cane J., Wood C.E. (2013). The behavior change technique taxonomy (v1) of 93 hierarchically clustered techniques: Building an international consensus for the reporting of behavior change interventions. Ann. Behav. Med..

[121] Murray C.J.L., Piot P. (2021). The Potential Future of the COVID-19 Pandemic: Will SARS-CoV-2 Become a Recurrent Seasonal Infection?. JAMA.

[122] Skegg D., Gluckman P., Boulton G., Hackmann H., Karim S.S.A., Piot P., Woopen C. (2021). Future scenarios for the COVID-19 pandemic. Lancet.

[123] Shanthanna H., Strand N.H., Provenzano D.A., Lobo C.A., Eldabe S., Bhatia A., Wegener J., Curtis K., Cohen S.P., Narouze S. (2020). Caring for patients with pain during the COVID-19 pandemic: Consensus recommendations from an international expert panel. Anaesthesia.

[124] Fu Y., Mcnichol E., Marczewski K., Closs J. (2016). Patient—Professional partnerships and chronic back pain self-management: A qualitative systematic review and synthesis. Health Soc. Care Community.

[125] Lewis Y.D., Elran-barak R., Tov R.G., Zubery E. (2021). The abrupt transition from face-to-face to online treatment for eating disorders: A pilot examination of patients’ perspectives during the COVID-19 lockdown. J. Eat. Disord..

[126] Schünemann H., Brożek J., Guyatt G., Oxman A. (2013). Manual GRADE para Calificar la Calidad de la Evidencia y la Fuerza de la Recomendación (1ª Ed. en Español). P.A Orrego & M.X. Rojas (Translator) March 2017. http://gdt.guidelinedevelopment.org/app/handbook/handbook.html.

